# Survival Models for Predictive Maintenance and Remaining Useful Life in Sensor-Enabled Smart Energy Networks: A Review

**DOI:** 10.3390/s26061915

**Published:** 2026-03-18

**Authors:** Mohammad Reza Shadi, Hamid Mirshekali, Maryamsadat Tahavori, Hamid Reza Shaker

**Affiliations:** 1 SDU Center for Energy Informatics, Maersk Mc-Kinney Moeller Institute, The Faculty of Engineering, University of Southern Denmark, 5230 Odense, Denmark; hmir@mmmi.sdu.dk (H.M.); hrsh@mmmi.sdu.dk (H.R.S.); 2 Section of Energy Technology and Computer Science, DTU Engineering Technology, Technical University of Denmark, 2750 Ballerup, Denmark; marta@dtu.dk

**Keywords:** asset management, censoring and truncation, Cox proportional hazards, learning-based survival models, random survival forest, RUL prediction

## Abstract

Smart energy networks, including electricity distribution and district heating, are increasingly operated as sensor-enabled infrastructures where maintenance decisions must be made under heterogeneous and time-varying operating conditions. In these settings, time-to-event data are rarely complete; preventive actions and limited observation horizons routinely introduce censoring and truncation, so models and validation procedures must account for partially observed lifetimes to avoid biased inference and misleading performance estimates. This review surveys survival models for predictive maintenance (PdM) and remaining useful life (RUL) estimation, spanning non-parametric, semi-parametric, parametric, and learning-based approaches, with emphasis on censoring-aware formulations and the use of static and time-varying covariates derived from sensor, inspection, and contextual information. A structured taxonomy and a systematic mapping of model families to data types, core assumptions (proportional hazards versus parametric distributional structure), and decision-oriented outputs such as risk ranking, horizon failure probabilities, and RUL distributions are presented. Evaluation practice is also synthesized by covering discrimination metrics, censoring-aware RUL accuracy measures, and probabilistic assessment via proper scoring rules, including the time-dependent Brier score and Integrated Brier Score (IBS). The review provides researchers and practitioners with a practical guide to selecting, fitting, and evaluating survival models for risk-informed maintenance planning in smart energy networks.

## 1. Introduction

Smart energy networks are shifting from schedule-based upkeep to data-driven reliability management. Electricity distribution networks and district heating networks (DHNs) now operate as sensor-rich systems where failures are costly, visible, and increasingly regulated. Operators must keep service reliable while assets age, operating conditions change, and digital control becomes more widespread. In this setting, maintenance is no longer only a technical task. It is a system-level decision problem with clear consequences for customers, reliability indices, and contractual compensation and penalties [1,2]. Medium-voltage assets are often a practical starting point because their age profile and failure history make them major drivers of outages [3].

Survival models offer a principled way to address this problem. They are designed for time-to-event prediction under censoring, which matches how grid and heating assets are observed in practice. They support risk estimation over time, not just binary fault flags. They can incorporate covariates from sensors, inspections, and context data. This makes them well suited for PdM and RUL estimation in real fleets. Yet applying survival analysis in smart energy networks is not straightforward. Data are heterogeneous, failures are rare, operating regimes drift, and maintenance actions change the risk process. Methods also need to be interpretable enough for engineering use and robust enough for operational deployment [4,5].

DHNs face a parallel reliability challenge, shaped by thermal and hydraulic constraints. They operate under high temperature and pressure in harsh environments. This exposes pipes and components to corrosion and leakage, with failures that can directly affect customer comfort and safety [6]. In DHN pipe systems, leaks dominate failure statistics and are often linked to corrosion or mechanical impacts. They are also widely regarded as the most severe form of damage among common degradation modes [7].

Historically, DHN operators could absorb early-stage problems by running the network hotter. Raising supply temperature can offset local losses, weak heat transfer, or gradual degradation and may delay visible service effects. The transition to low-temperature district heating, often framed as fourth-generation DH, reduces this operational buffer. When systems are designed to run closer to the minimum temperature required at the customer level, there is less margin to mask emerging faults through temperature increases. As a result, degradation is more likely to appear quickly as measurable and noticeable service shortfalls, such as insufficient heat delivery, rather than remaining hidden until a later failure [8].

For distribution system operators (DSOs), maintenance is fundamentally a resource-constrained risk allocation problem. DSOs must determine which medium-voltage assets to repair or replace, when to intervene, and where residual risk can be tolerated, while the consequences are directly reflected in continuity of supply and regulatory performance. What makes this difficult in practice is that asset condition evidence is partial and delayed (many components never fail within the observation window), and failure processes are non-stationary (loading, switching, climate, and network reconfiguration change the stress landscape). Interventions alter the time-to-failure process itself, replacement resets effective age, whereas repairs usually produce partial risk reduction and can shift the dominant failure location rather than eliminating risk [9,10].

Accordingly, DSOs require models that represent risk as a time-dependent quantity and remain practical for planning. Such models should generate comparable and updateable risk trajectories across heterogeneous fleets so that renewal prioritization, intervention timing, and portfolio-level impacts can be justified in operational and regulatory terms. Survival models align well with this decision interface because they are designed for time-to-event analysis under censoring and provide interpretable measures such as hazard and survival. These outputs also support aggregation from individual assets to feeder- and system-level assessments [11,12].

This operational reality is what drives the move from age-threshold policies to PdM and RUL estimation in both DHN and medium-voltage (MV) distribution asset management. Fixed lifetime assumptions and simple age-based replacement do not provide a defensible basis for prioritization when risk differs sharply across nominally similar assets. The planning problem therefore becomes identifying when risk crosses an unacceptable level for each asset class so that replacement, refurbishment, or intensified monitoring can be scheduled before outages and service deterioration occur, and so that limited budgets are allocated to interventions with measurable reliability impact [13,14].

The transition toward PdM and RUL estimation depends on what the network can reliably observe. Sensor and inspection practices determine which degradation mechanisms become measurable and can be used as covariates in reliability and RUL models. In MV cable fleets, condition assessment often relies on diagnostic tests that provide information beyond installation age, including very low frequency dissipation factor (tan δ) [15], partial discharge measurements [16], and oversheath testing [17,18]. These measurements translate latent insulation degradation into observable indicators that support risk ranking and intervention scheduling.

Observability also differs across asset classes, which influences how survival models are applied in practice. For distribution transformers, internal sensing is often limited at scale. As a result, risk and RUL estimation may rely on indirect signals such as loading inferred from smart meters and ambient temperature as proxies for thermal stress and aging [19,20]. In DHNs, available data can be broader, including process measurements such as temperature, pressure, and flow, inspection records, and leakage detection. However, monitoring coverage varies widely across systems, and many networks still lack continuous measurements along pipe segments [21].

Table 1 indicates that recent reviews in prognostics and health management and data-driven RUL prediction largely emphasize deep-learning RUL pipelines or domain-specific PdM applications, while giving limited attention to censoring/truncation mechanisms and to a coherent taxonomy of survival model families. This review therefore focuses on survival (time-to-event) models for PdM and RUL estimation in sensor-enabled smart energy networks, encompassing electricity distribution networks and district heating networks. Non-parametric, semi-parametric, parametric, and learning-based approaches are synthesized, and these families are mapped to the data structures and assumptions they require (e.g., static versus time-varying covariates, and censoring-aware likelihoods) and to the operational outputs used in maintenance planning (risk ranking, horizon failure probabilities, and RUL distributions). In addition, evaluation practice is consolidated by explicitly pairing discrimination with censoring-aware RUL accuracy measures and proper scoring rules such as the time-dependent Brier score and Integrated Brier Score to assess both ranking and probabilistic calibration. The resulting synthesis supports reproducible method selection for researchers and defensible, risk-informed maintenance planning for practitioners.

## 2. Taxonomy of Survival Models

**Figure 1:**
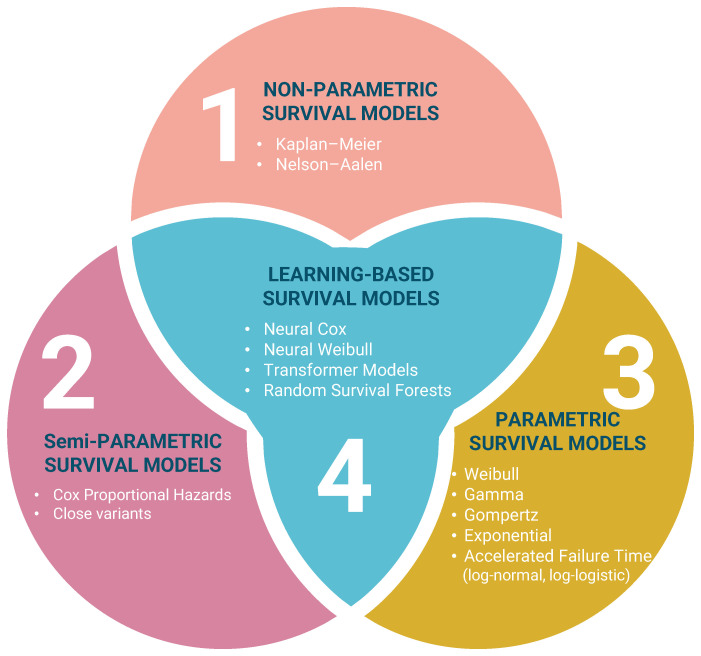
Taxonomy of survival models for predictive maintenance and asset management.

This time-dependent view of failure risk is well established in infrastructure reliability and is commonly addressed using a limited number of survival model families. Figure 1 summarizes the main taxonomy adopted in this review, namely non-parametric, semi-parametric, parametric, and learning-based survival models. These approaches have been widely used for applications such as pipe-break risk assessment and renewal prioritization [34], while related Weibull-based reliability models have also been applied in power distribution to represent time-varying component failure probabilities [2]. This taxonomy provides the conceptual structure for the methodological discussions in the following sections.

Within this taxonomy, the principal distinction lies in the extent of assumptions imposed on the time-to-event process and in the way covariate effects are handled. Non-parametric models, such as Kaplan–Meier and Nelson–Aalen, describe survival behaviour directly from observed event data with minimal structural assumptions and are therefore useful for baseline estimation and descriptive comparison. Semi-parametric models, represented mainly by the Cox proportional hazards model and its close variants, preserve flexibility in the baseline hazard while enabling the analysis of covariate effects. Parametric survival models, including Weibull, Gamma, Gompertz, Exponential, and accelerated failure time formulations such as log-normal and log-logistic models, specify an explicit lifetime distribution and are especially useful when hazard-shape interpretation or extrapolation is required. Learning-based survival models further extend this taxonomy by capturing non-linear effects and higher-dimensional interactions through approaches such as neural Cox, neural Weibull, transformer-based survival models, and random survival forests.

A further advantage of this taxonomy is that it helps align model choice with the characteristics of available data and the intended maintenance objective. In practice, asset-management problems may differ substantially in terms of sample size, censoring structure, covariate richness, and the need for interpretability versus predictive flexibility. As a result, the selection of a survival modelling approach is rarely universal, but instead depends on whether the goal is descriptive reliability assessment, covariate effect analysis, long-horizon lifetime estimation, or data-driven risk prediction. Framing the literature through this taxonomy therefore supports a more systematic comparison of methods and clarifies the contexts in which different survival-model families are most suitable.

## 3. Sensors and Censoring

In survival and reliability studies, censoring occurs when an asset is observed but its exact event time is not known. The record still contains valid information because the asset is known to have survived up to a specific time. This is common in maintenance data where observation ends before many failures occur, or where preventive maintenance stops follow-up before failure and creates right-censored records [10]. In condition-based maintenance datasets, this appears explicitly as a mix of failure histories and suspension histories, where components are replaced preventively rather than failing [35]. By contrast, truncation refers to a selection mechanism in which some lifetimes are not observed because an asset is included in the dataset only if its event time falls within a predefined observation window. As a result, failures occurring outside the window are absent from the sample, which changes the composition of the observed population and can bias inference if not handled explicitly [36]. For instance, a power transformer fleet monitored from 1980 onward includes only units that were still in service in 1980. Transformers installed and failing before 1980 are absent from the dataset.

**Figure 2:**
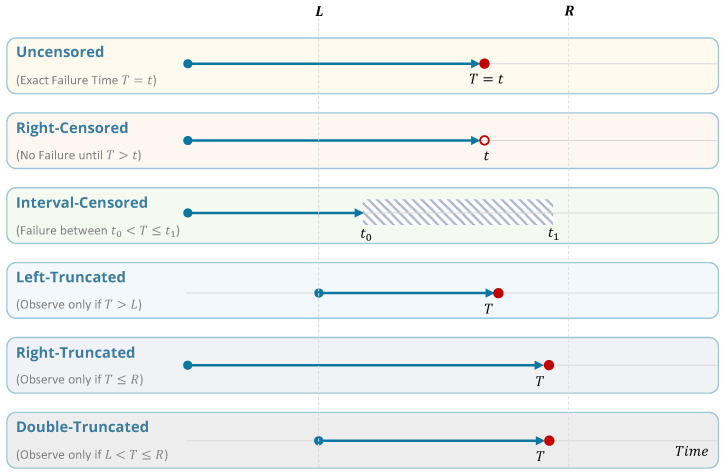
Common censoring and truncation types in survival data.

Let *T* denote the true event (failure) time, *t* an observed time point (either an exact event time or a censoring time), and *L* and *R* the left and right truncation limits that define the study’s inclusion window (Figure 2).

- **Uncensored:** exact failure time is known (T=t).
- **Right-censored:** failure has not occurred by the last observation (T>t).
- **Interval-censored:** failure occurred within a known time window ($t_{0}<T\leq t_{1}$).
- **Left-truncated:** units that failed before entry are unobserved; only those with T>L appear.
- **Right-truncated:** units with very late failures are unobserved; only those with T≤R appear.
- **Double-truncated:** only units with failures within the inclusion window are observed (L<T≤R) [37].

Left truncation deserves particular attention in smart energy asset datasets because many components have already been in service for years before digital monitoring begins. In such settings, the observed sample is conditioned on survival up to the monitoring entry time, which can bias hazard and survival estimates if this delayed entry is ignored. The standard treatment is to use delayed-entry survival modeling, where an asset contributes to the risk set only after its observation start time rather than from installation. In semi-parametric models such as Cox PH, this is handled through entry-time-adjusted risk sets, while in parametric models the likelihood is conditioned on survival up to the truncation time. For learning-based survival models, the same principle should be preserved in the training objective and sampling logic so that prediction targets remain consistent with the observed at-risk population. When installation dates or historical operating records are incomplete, additional bias mitigation may require asset-registry reconstruction, use of inspection and replacement records, and sensitivity analysis under alternative assumptions on unobserved early-life exposure. In practice, careful treatment of left truncation is especially important in legacy power and district heating fleets, where sensor deployment often starts long after commissioning.

Beyond defining censoring and truncation, sensors play a central role in survival and RUL modeling by providing time-varying evidence of operating stress and incipient abnormal behavior. This allows failure risk to be conditioned on more than age and installation metadata. In electricity distribution systems, SCADA and distribution automation supply continuous measurements of system state and loading, including current, voltage, power, and switch status. These signals can be translated into asset-level exposure and incorporated as covariates that modulate hazard over time. In district heating networks, supervisory monitoring similarly captures temperature, pressure, flow rates, and energy-related variables across substations and network segments, enabling models to represent hydraulic and thermal operating regimes rather than treating failures as purely age-driven. In this way, sensor-based condition evidence converts operational variability into measurable covariates that explain heterogeneity in failure risk and support risk updates as operating conditions evolve [38,39].

Sensor data can also change which outcomes are observed. When monitoring systems flag abnormal conditions, operators often replace an asset before it fails. In the dataset, this replacement ends the observation period without a recorded failure time. The record is therefore right-censored; the asset is known to have survived up to the replacement time, but when failure would have occurred is not observed. If many assets are preventively replaced, the dataset contains fewer run-to-failure examples, which reduces the amount of direct failure information available for learning and evaluation. This censoring mechanism must be modeled explicitly in the survival likelihood and reflected in the evaluation protocol [40,41].

Data characteristics differ substantially between electricity and district heating assets, and these differences affect how survival models should be formulated. In power systems, assets such as cables and transformers are often monitored through a combination of lower-rate SCADA signals, periodic diagnostic tests, and indirect stress proxies, while failures may arise from insulation degradation, thermal aging, switching stress, or partial discharge activity. In DHNs, the data more commonly reflect thermal–hydraulic operation through temperature, pressure, flow, and leak-related observations, with degradation dominated by corrosion, leakage, and external mechanical impacts. These differences imply distinct sampling structures, noise sources, and observability levels. Accordingly, survival models for power assets often rely more on static covariates, inspection features, and sparse time-varying updates, whereas DHN applications can more naturally exploit process-oriented covariates and operating-regime information. In both cases, model design should follow the sensing reality of the asset class, including the choice of covariates, update frequency, censoring treatment, and level of model complexity.

## 4. Survival Models

**Figure 3:**
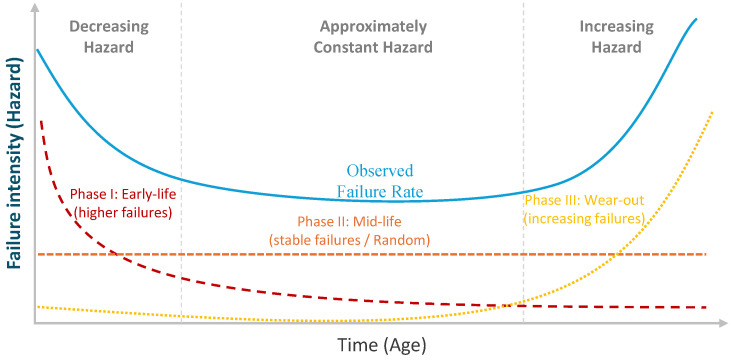
Bathtub-shaped hazard curve illustrating three lifecycle phases.

The practical value of survival models in utility maintenance planning comes from how naturally their predictions translate into action. Utilities rarely need an abstract “health score” on its own. They need forecasts that answer planning questions in the same units as their decisions: time and risk. Survival modeling provides that interface by estimating the likelihood that an asset will remain in service over a defined horizon, such as the next *k* years. It also describes how failure pressure changes with age through the hazard function. This hazard can change with time and may not move in only one direction. This is often described by the bathtub curve [42], as illustrated in Figure 3:

- **Early-life stage (higher failures):** failures are relatively common due to installation issues or early defects.
- **Mid-life stage (stable failures):** the failure rate becomes fairly steady for a long period.
- **Late-life stage (increasing failures):** failures rise again as aging and wear-out dominate.

Together, these outputs allow planners to compare assets with different ages and histories on a common basis. They also support transparent intervention rules, for example scheduling renewal when the projected near-term risk crosses a policy threshold.

To make this decision interface explicit, the PdM and RUL prediction tasks are formulated in terms of the survival function, hazard rate, and conditional remaining-life distribution. In this way, the mapping from survival-model outputs to horizon failure probabilities, RUL summaries, and threshold-based maintenance decisions is clarified.

### 4.1. Problem Formulation: Predictive Maintenance and RUL Estimation

PdM aims to forecast future failure behavior and convert these forecasts into actionable maintenance decisions. Let *T* denote the (random) failure time of an asset. The survival function

(1) S(t)=Pr(T>t),

gives the probability that the asset remains operational beyond time *t*. The hazard function

(2) $$h\left(t\right)=\lim_{\Delta t\to 0}\frac{\mathrm{Pr}\;\left(t\leq T<t+\Delta t|T\geq t\right)}{\Delta t},$$

describes the instantaneous failure intensity at time *t*, and the cumulative hazard

(3) $$H\left(t\right)=\int_{0}^{t}h\left(u\right)\;du$$

links to the survival function through

(4) $$S\left(t\right)=\exp\;\left[-H(t)\right].$$

#### 4.1.1. Remaining Useful Life

Given that an asset has survived up to the current time $t_{0}$, the RUL is the conditional random variable

(5) $$\mathrm{RUL}\left(t_{0}\right)=\left(T-t_{0}\right)|\left(T>t_{0}\right).$$

A survival model provides the conditional survival of the remaining life via

(6) $$\mathrm{Pr}\left(\mathrm{RUL}\left(t_{0}\right)>u\right)=\mathrm{Pr}\left(T>t_{0}+u|T>t_{0}\right)=\frac{S(t_{0}+u)}{S(t_{0})},\;u\geq 0,$$

which yields the expected remaining life (when finite) as

(7) $$E\left[\mathrm{RUL}\left(t_{0}\right)\right]=\int_{0}^{\infty }\mathrm{Pr}\left(\mathrm{RUL}\left(t_{0}\right)>u\right)\;du=\int_{0}^{\infty }\frac{S(t_{0}+u)}{S(t_{0})}\;du.$$

In practice, it is also common to report RUL quantiles obtained from the conditional survival curve, where q∈(0,1) denotes the desired quantile level (q=0.5 for the median), i.e., find $u_{q}$ such that

(8) $$\frac{S(t_{0}+u_{q})}{S(t_{0})}=1-q.$$

#### 4.1.2. Horizon Failure Probability

For a planning horizon τ>0, the probability of failure within $\left(t_{0},t_{0}+\tau \right]$ conditional on survival up to $t_{0}$ is

(9) $$\mathrm{Pr}\left(t_{0}<T\leq t_{0}+\tau |T>t_{0}\right)=1-\mathrm{Pr}\left(T>t_{0}+\tau |T>t_{0}\right)=1-\frac{S(t_{0}+\tau )}{S(t_{0})}.$$

Survival-based PdM models support decision-making through (i) risk ranking using h(t∣z) or a risk score, (ii) horizon failure probabilities over planning windows, and (iii) RUL distributions or their summaries (mean/quantiles). A common risk-informed intervention rule can be written as

(10) $$\mathrm{Intervene}\;\mathrm{if}\;\;\mathrm{Pr}(t_{0}<T\leq t_{0}+\tau |T>t_{0})>\gamma ,$$

where γ∈(0,1) is a policy threshold reflecting operational constraints, reliability targets, and risk tolerance. This formulation explicitly connects survival outputs (S(t), h(t), H(t)) to PdM decisions and RUL estimation.

While all survival approaches aim to estimate the same decision-relevant quantities (the survival function S(t), hazard h(t), and cumulative hazard H(t)) the taxonomy used here is defined by the degree of structural assumptions imposed on the time-to-event process and the way covariates are incorporated. Non-parametric models estimate S(t) or H(t) directly from observed event/risk sets with minimal distributional assumptions and typically lack a general regression form for continuous covariates (beyond stratification). Semi-parametric models introduce a parametric covariate effect (hazard ratios) while leaving the baseline hazard unspecified, enabling covariate-aware risk ranking without assuming a full lifetime distribution. Parametric models assume an explicit distributional form for *T*, yielding closed-form S(t) and h(t) and supporting absolute risk estimation and extrapolation. Learning-based survival models retain censoring-aware objectives and survival outputs but replace linear predictors or fixed functional forms with flexible function approximators (e.g., trees or neural networks), improving capacity to capture non-linearities and interactions at the cost of higher data requirements and potentially reduced interpretability.

### 4.2. Non-Parametric Survival Models

Non-parametric survival models make minimal assumptions about the underlying failure mechanism and estimate survival probabilities or hazard rates directly from observed data, without specifying a particular lifetime distribution [43]. In the following, different non-parametric survival models are reviewed.

#### 4.2.1. Kaplan–Meier

The Kaplan–Meier (KM) estimator, also known as the product-limit estimator, is the most commonly used non-parametric method for estimating the survival function [44,45]. It calculates the survival probability S(t) by sequentially multiplying the chances of surviving each observed failure time, given that the asset was still operational just before that time. Let $t_{\left(1\right)}<t_{\left(2\right)}<\ldots <t_{\left(K\right)}$ denote the ordered event times, where $e_{j}$ failures occur among $r_{j}$ assets at risk at time $t_{\left(j\right)}$. The KM estimator is defined as

(11) $$\hat{S}\left(t\right)\;=\;\prod_{t_{\left(j\right)}\leq t}\left(1-\frac{e_{j}}{r_{j}}\right)$$

The resulting estimator yields a stepwise survival curve that decreases only at observed failure times [46]. Right-censored observations, for which the failure time is unknown beyond the study horizon, are handled by reducing the risk set $r_{j}$ at subsequent times, without contributing an event. As a result, censoring does not produce drops in the survival curve but influences the estimate by shrinking the effective sample size [47].

The KM estimator is widely used as a baseline tool in survival analysis, offering an empirical summary of time-to-failure distributions and enabling comparisons across asset categories. In PdM and asset management, stratified KM curves are often used to benchmark component reliability across subgroups, for example, comparing failure behavior across different cable types, materials, or operating environments [48]. However, because the estimator does not model covariates, it requires separate curves for each group and cannot accommodate continuous sensor readings or multivariate feature sets.

#### 4.2.2. Nelson–Aalen

Another non-parametric approach is the Nelson–Aalen (NA) estimator, which focuses on the cumulative hazard function H(t) rather than S(t). The NA estimator accumulates hazard increments over time:

(12) $$\hat{H}\left(t\right)=\sum_{t_{\left(j\right)}\leq t}\frac{e_{j}}{r_{j}}$$

where $e_{j}$ is the number of failures and $r_{j}$ the number at risk at time $t_{\left(j\right)}$. This estimator is based on a counting-process framework and, on average, gives an accurate estimate of the cumulative failure risk [49]. Once $\hat{H}\left(t\right)$ is obtained, the corresponding survival estimate can be computed via the exponential transformation

(13) $$\hat{S}\left(t\right)=\exp\left[-\hat{H}\left(t\right)\right]$$

This formulation links the NA estimator to the KM estimator, which expresses survival as the product of conditional survival probabilities:

(14) $$\hat{S}\left(t\right)=\prod \left(1-\frac{e_{j}}{r_{j}}\right)$$

When the hazard increments $\frac{e_{j}}{r_{j}}$ are small, this product can be approximated using the first-order Taylor expansion log(1−x)≈−x, yielding

(15) $$\hat{S}\left(t\right)\approx \exp\left(-\sum \frac{e_{j}}{r_{j}}\right)$$

This approximation reveals the connection between the KM and NA estimators. Both are non-parametric methods that use the same event and risk information but represent it through survival probability and cumulative hazard, respectively [50]. In PdM, the NA estimator offers a complementary view by quantifying the accumulated risk over time, which can support reliability assessments and maintenance planning. Like KM, it does not rely on parametric assumptions, making it useful for initial exploration of failure patterns. However, its inability to incorporate covariates limits its use in individualized prediction. This motivates the need for regression-based survival models that can link asset-specific features, such as sensor measurements or design parameters, to failure risk for more precise RUL estimation.

### 4.3. Semi-Parametric Survival Models

Semi-parametric survival models provide a flexible framework for incorporating covariate information without fully specifying the functional form of the underlying hazard or survival distribution. The most widely used model in this class is the Cox proportional hazards (PH) model [51], which separates time-dependent baseline risk from covariate effects. The remainder of this section reviews key semi-parametric survival models.

#### 4.3.1. Cox Proportional Hazards

In this model, the hazard function for an asset with covariate vector *z* is expressed as the product of two components:

(i) an unspecified baseline hazard $h_{0}\left(t\right)$, which describes how failure risk changes over time for a baseline (reference) asset;
(ii) a parametric term $\exp(\beta^{⊤}z)$, which quantifies how covariates proportionally shift the hazard relative to the baseline.

Formally, the Cox proportional hazards model is written as

(16) $$h\left(t|z\right)=h_{0}\left(t\right)\exp\left(\beta^{⊤}z\right)$$

This proportional hazards structure implies that covariates shift the risk of failure by a constant factor over time. For example, if $\beta_{1}$ corresponds to a temperature effect, $\exp(\beta_{1})$ represents the constant hazard ratio for a one-unit increase in temperature. Cox’s semi-parametric approach thus addresses a key limitation of KM by enabling covariate modeling (e.g., sensor measurements, operational conditions) [1], while still not assuming a specific lifetime distribution for baseline risk.

#### 4.3.2. Partial Likelihood for Cox

Model coefficients β are typically estimated via partial likelihood, which maximizes the hazard discrimination between failed and surviving units at each event time without needing to specify $h_{0}\left(t\right)$. This approach yields estimates of covariate effects (interpreted as hazard ratios) independently of the baseline risk. Although the baseline hazard is not required during model fitting, it can still be estimated afterward if needed—commonly using methods like Breslow’s estimator or by smoothing the Nelson–Aalen cumulative hazard. The main advantage of Cox regression lies in this separation; it avoids assumptions about the shape of the hazard over time while allowing practitioners to identify which covariates influence failure risk [34].

#### 4.3.3. Time-Varying Covariates for Cox (Extended)

Cox’s model can be extended to accommodate covariates z(t) that evolve over time, such as operating temperature, load, or vibration level. In this formulation, the hazard at time *t* depends on the current values of both time-varying and static covariates and is given by

(17) $$h\left(t|z\left(t\right)\right)=h_{0}\left(t\right)\exp\left(\sum_{j=1}^{p_{1}}\delta_{j}z_{j}\left(t\right)+\sum_{i=1}^{p_{2}}\beta_{i}z_{i}\right)$$

where $z_{j}\left(t\right)$ are time-dependent covariates with corresponding coefficients $\delta_{j}$, and $z_{i}$ are time-invariant (static) covariates with coefficients $\beta_{i}$. This formulation, often referred to as the extended Cox model, allows the hazard ratio between two units to change over time [52]. Such flexibility is particularly important in PdM, where asset condition indicators such as oil quality, pressure, or temperature may vary during operation and influence risk dynamically, while the inclusion of time-varying covariates technically violates the proportional hazards assumption in its strict form when $z_{j}\left(t\right)$ changes over time [38,53], the model can still be estimated using a modified version of partial likelihood and remains widely applicable in practice.

The Cox model and its extensions are widely used in asset management for their interpretability and flexibility. By examining the sign and magnitude of β coefficients (or hazard ratios $e^{\beta }$), one can identify which factors most strongly increase failure risk, information crucial for maintenance decision-making [54,55]. For example, a Cox model can indicate that higher load or longer operating hours increases the hazard by a multiplicative factor, which can help engineers prioritize critical operating conditions. A key limitation, however, is the proportional hazards (PH) assumption: each covariate is assumed to have a time-invariant effect on the hazard. This can be restrictive in PdM settings, where the impact of variables such as vibration or temperature may intensify as an asset ages.

Table 2 summarizes the core formulas for the non-parametric and semi-parametric survival models discussed in this section.

### 4.4. Parametric Survival Models

Parametric survival models assume a specific probability distribution for failure times *T*, yielding explicit formulas for the survival function S(t) and hazard h(t). In reliability engineering and asset management, parametric models are common because many components’ lifetime behaviors can be approximated by standard distributions, and having a closed-form model allows direct calculation of quantities like mean time to failure or failure probability by a given age [56]. Parametric models are commonly specified either as PH models with a parametric baseline hazard or as Accelerated Failure Time (AFT) models; for some families (notably Weibull), these representations are closely related.

Beyond the Weibull distribution, several other parametric specifications are frequently used in PdM, each encoding a different assumption about how failure risk evolves over time. The exponential model is the simplest case (corresponding to a constant hazard) and is often used as a baseline when data are sparse or when a memoryless failure mechanism is plausible. In contrast, log-normal and log-logistic models are commonly expressed in an AFT form and can represent non-monotonic hazard patterns, where risk increases and later decreases with age, a behavior that a Weibull hazard (which is monotone in *t*) cannot capture. Gompertz models are also used in reliability contexts to represent exponentially increasing (or decreasing) hazards over time. In general, the choice of distribution is guided by empirical hazard trends and goodness-of-fit considerations. When the assumed form is appropriate, parametric models tend to be statistically efficient and facilitate closed-form estimation of failure probabilities. Moreover, they naturally support extrapolation beyond the observed time range, enabling long-horizon survival prediction for asset management and scenario analysis [57].

#### 4.4.1. Weibull Distribution

The Weibull distribution is one of the most widely used parametric models in reliability and PdM due to its flexibility in capturing various failure behaviors. It is defined by two positive parameters: a shape parameter α>0 and a scale parameter λ>0. The hazard and survival functions for the Weibull distribution are given by:

(18) $$\begin{array}{cc} h(t) & =\frac{\alpha }{\lambda }{\left(\frac{t}{\lambda }\right)}^{\alpha -1} \end{array}$$

(19) $$\begin{array}{cc} S(t) & =\exp\left[-{\left(\frac{t}{\lambda }\right)}^{\alpha }\right],\;t\geq 0 \end{array}$$

**Figure 4:**
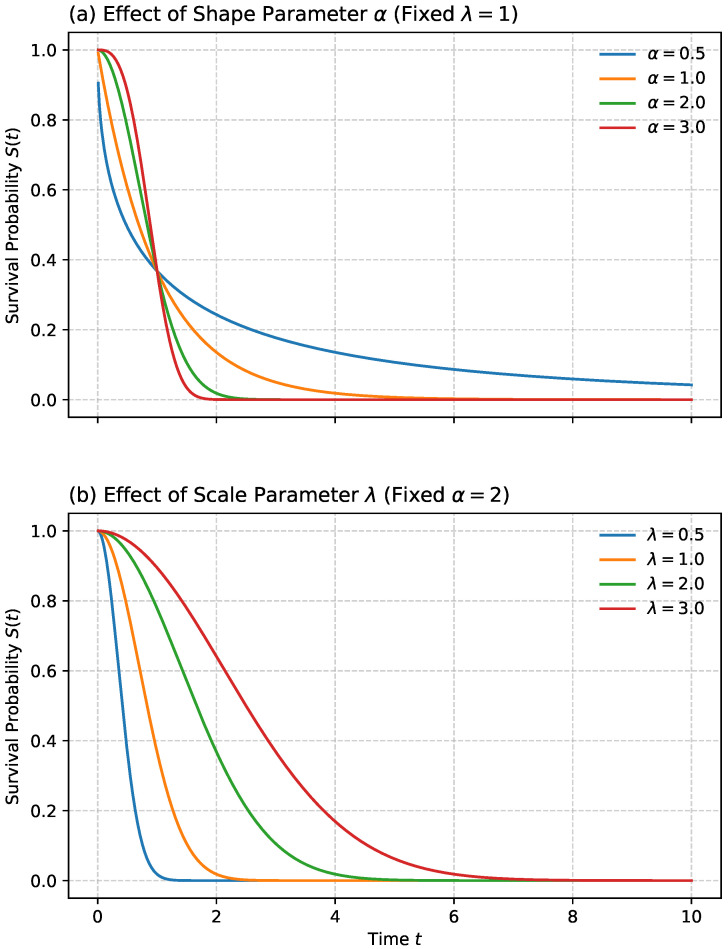
Weibull survival functions illustrating the effect of (**a**) shape parameter α and (**b**) scale parameter λ on asset lifetime distributions.

The shape parameter α determines the nature of the hazard rate: α>1 implies an increasing hazard over time (e.g., wear-out failures), α<1 indicates a decreasing hazard, and α=1 corresponds to a constant hazard, reducing the model to the exponential case (as shown in Figure 4). These properties make the Weibull distribution highly suitable for modeling asset failures across different lifecycle stages [58,59,60].

The Weibull model can also be expressed in the AFT form, where covariates have a multiplicative effect on failure time:

(20) $$\ln T=\beta^{⊤}z+\sigma \epsilon$$

where *z* is the covariate vector, β is the coefficient vector, σ is a scale parameter, and ϵ follows an extreme value distribution. In this formulation, a unit increase in $z_{j}$ accelerates or decelerates the failure time by a factor of $\exp(\beta_{j})$. Equivalently, the scale parameter λ can be modeled as a function of covariates:

(21) $$\lambda \left(z\right)=\exp\left(-\beta^{⊤}z\right),\;\mathrm{so}\;\mathrm{that}\;S\left(t|z\right)=\exp\left[-{\left(\frac{t\;e^{-\beta^{⊤}z}}{\lambda_{0}}\right)}^{\alpha }\right]$$

where $\lambda_{0}$ is a baseline scale parameter. Larger values of $\beta^{⊤}z$ correspond to shorter effective lifetimes.

Alternatively, the Weibull model can be embedded in a proportional hazards (PH) framework by specifying a Weibull baseline hazard function $h_{0}\left(t\right)$ and incorporating covariate effects via a log-linear hazard ratio:

(22) $$h\left(t|z\right)=h_{0}\left(t\right)\exp\left(\beta^{⊤}z\right),\;\mathrm{with}\;h_{0}\left(t\right)=\frac{\alpha }{\lambda }{\left(\frac{t}{\lambda }\right)}^{\alpha -1}$$

This dual interpretability, through both AFT and PH formulations, makes the Weibull distribution especially attractive in practice, allowing practitioners to flexibly model how asset characteristics influence failure time or risk. Even in the absence of detailed sensor data, the Weibull model remains valuable for capturing general aging trends or early-life failure patterns directly from observed failure times [61,62].

#### 4.4.2. Exponential Distribution

The exponential distribution is a special case of the Weibull model with shape parameter α=1, yielding a constant hazard rate:

(23) $$h\left(t\right)=\frac{1}{\lambda }$$

and the corresponding survival function

(24) $$S\left(t\right)=\exp\;\left(-\frac{t}{\lambda }\right)$$

Here, λ is the scale parameter (equal to the mean lifetime) and 1/λ is the constant failure rate. This specification implies a memoryless failure process, meaning the instantaneous risk of failure does not depend on the asset age. Although a constant hazard is often unrealistic for components that wear out with age, the exponential model is still useful as a simple baseline, and, in asset management, corresponds to assuming a constant annual failure rate [63].

#### 4.4.3. Gompertz Distribution

The Gompertz distribution models a hazard rate that changes exponentially with time. It is commonly parameterized by *a* and *b* via

(25) $$h\left(t\right)=b\;e^{at}$$

so that the failure rate increases over time if a>0 and decreases if a<0 [64]. Integrating the hazard yields the survival function

(26) $$S\left(t\right)=\exp\;\left[-\frac{b}{a}\left(e^{at}-1\right)\right],\;a\neq 0$$

When a→0, the Gompertz model reduces to a constant-hazard form, and the survival function becomes S(t)=exp(−bt), which is the exponential model. In PdM applications, the Gompertz distribution is therefore a natural choice when observed failure patterns indicate that hazard grows rapidly with age, such as when assets exhibit strongly accelerating wear after a certain service duration [65].

#### 4.4.4. Log-Normal Distribution (AFT)

In the log-normal model, the logarithm of failure time is assumed Gaussian. In an AFT formulation,

(27) $$\ln T=\beta^{⊤}z+\sigma \epsilon$$

where *z* denotes covariates, β is the coefficient vector, and σ is a scale parameter, and ϵ is a standard normal random variable, ϵ∼N(0,1). The survival function can be written as

(28) $$S\left(t\right)=1-\Phi \;\left(\frac{\ln t-\mu }{\sigma }\right)$$

where Φ(·) is the standard normal cumulative distribution function. A characteristic feature of the log-normal model is a non-monotonic hazard shape: the hazard typically starts near zero, rises to a peak, and then decreases at later ages. This makes the distribution suitable for mechanisms where risk builds up and later attenuates, or where time-to-failure exhibits substantial variability and long tails [66,67,68].

#### 4.4.5. Log-Logistic Distribution (AFT)

The log-logistic model assumes lnT follows a logistic distribution and provides closed-form expressions for both survival and hazard. With shape parameter α>0 and scale parameter 1/λ, the survival function is

(29) $$S\left(t\right)=\frac{1}{1+{\left(\lambda t\right)}^{\alpha }}$$

and the hazard function is

(30) $$h\left(t\right)=\frac{\lambda \alpha t^{\alpha -1}}{1+{\left(\lambda t\right)}^{\alpha }}$$

The hazard increases and then decreases when α>1 (unimodal hazard) and is decreasing for α≤1 [69]. This flexibility is useful in reliability settings where risk peaks at intermediate ages. In an AFT interpretation, covariates shift the time scale of failure through Equation (27), where ϵ follows a logistic distribution, implying that features *z* accelerate or decelerate the lifetime by rescaling time rather than directly modifying a baseline hazard.

#### 4.4.6. Gamma Distribution

The Gamma distribution is another flexible parametric choice for modeling failure time T≥0. It is defined by two positive parameters: a shape parameter κ and a scale parameter θ. The probability density function is

(31) $$f\left(t\right)\;=\;\frac{1}{\Gamma \left(\kappa \right)\;\theta^{\kappa }}\;t^{\kappa -1}\exp\;\left(-\frac{t}{\theta }\right)\;t\geq 0,$$

where Γ(·) denotes the Gamma function. The cumulative distribution function is

(32) $$F\left(t\right)\;=\;\frac{\gamma (\kappa ,\;t/\theta )}{\Gamma (\kappa )}$$

The corresponding survival function is

(33) $$S\left(t\right)\;=\;1-F\left(t\right)\;=\;\frac{\Gamma (\kappa ,\;t/\theta )}{\Gamma (\kappa )}$$

where γ(·,·) and Γ(·,·) denote the lower and upper incomplete Gamma functions, respectively. As in other parametric survival models, the hazard function is obtained from

(34) $$h\left(t\right)\;=\;\frac{f(t)}{S(t)}.$$

In practical terms, the Gamma model is useful because the shape parameter κ changes how failures concentrate over time. For example, κ=1 reduces the model to the exponential case (constant hazard), while κ≠1 allows more flexible lifetime shapes. This makes the Gamma distribution a reasonable alternative when empirical failure-time patterns are not well captured by a Weibull shape, yet a compact two-parameter lifetime model is still preferred [70].

**Figure 5:**
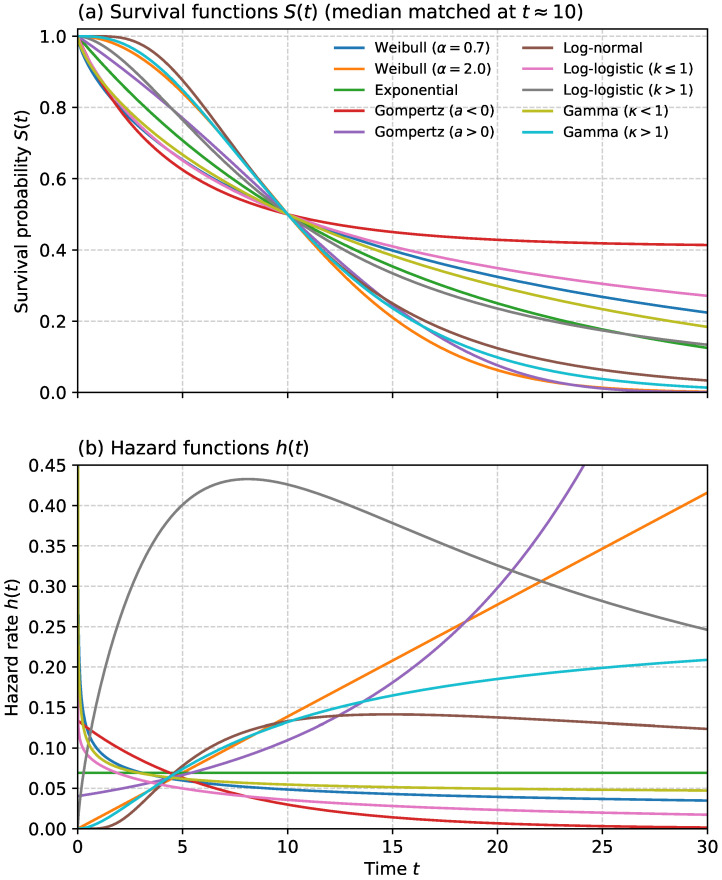
Comparison of common parametric survival models: (**a**) survival functions S(t) calibrated to a common median lifetime (S(10)≈0.5); (**b**) corresponding hazard functions h(t) illustrating constant, monotone, and peaked risk regimes.

Figure 5 provides a side-by-side comparison of the parametric survival models described in this subsection. In panel (a), all distributions are calibrated to have the same median lifetime of approximately 10 years, i.e., S(10)≈0.5 for every curve; this makes the models directly comparable and highlights differences in early-life behavior and long-term tails. Panel (b) shows the corresponding hazard functions h(t), which make the underlying risk assumptions explicit: the exponential model implies a constant hazard, Weibull and Gompertz variants produce monotone hazards (decreasing for early-life failure regimes and increasing for wear-out or accelerating aging), while log-normal and log-logistic models allow a peaked hazard that increases to a maximum at intermediate ages and then declines. The Gamma model provides an additional parsimonious alternative, with κ<1 yielding decreasing hazard, and κ>1 yielding increasing hazard. Overall, Figure 5 emphasizes that selecting a parametric distribution amounts to selecting a hazard-shape assumption, which directly influences extrapolated survival and maintenance planning.

### 4.5. Learning-Based Survival Models

Learning-based survival models generalize the preceding non-parametric, semi-parametric, and parametric families by replacing rigid predictors (e.g., linear Cox risk scores) or fixed lifetime forms with flexible function approximators such as tree ensembles and neural networks, while still producing survival quantities of direct operational value in PdM, including h(t∣z), H(t∣z), and S(t∣z) [71]. Their central promise is to learn non-linear effects and higher-order interactions from heterogeneous covariates (asset metadata, environment, and sensor-derived indicators) that are difficult to encode in classical models, yet without sacrificing the time-to-event interpretation that planners need for horizon risk and RUL estimation. Crucially, these models must be trained in a censoring-aware manner: censored units still carry information (they survived up to their last observation), and ignoring this structure can bias risk estimation and miscalibrate RUL forecasts. In practice, censoring-awareness is enforced either through Cox-type objectives (partial likelihood, as in neural Cox/DeepSurv) or through direct maximization of a likelihood induced by an explicit survival parameterization (as in neural parametric and hybrid models) [72].

For right-censored data, these models share a common likelihood formulation that combines information from both observed failures and censored observations. Intuitively, each asset contributes exactly what is known from the data: if it fails, the model should assign high probability to failing around that time; if it does not fail within the observation window, the model should assign high probability to surviving at least up to the last observed time. Let $t_{i}$ denote the observed time for unit *i* (the failure time if the event occurs, or the last observation time if the unit is censored), and let $\delta_{i}\in \left\{0,1\right\}$ be the event indicator, with $\delta_{i}=1$ if a failure is observed and $\delta_{i}=0$ if the unit is right-censored. Then, under conditional independence, the joint likelihood can be written as:

(35) $$L\left(\theta \right)=\prod_{i=1}^{n}f{\left(t_{i}|z_{i};\theta \right)}^{\delta_{i}}\;S{\left(t_{i}|z_{i};\theta \right)}^{1-\delta_{i}}$$

and its log form

(36) $$\ell \left(\theta \right)=\sum_{i=1}^{n}\left[\delta_{i}\log f\left(t_{i}|z_{i};\theta \right)+\left(1-\delta_{i}\right)\log S\left(t_{i}|z_{i};\theta \right)\right]$$

Conceptually, failures ($\delta_{i}=1$) pull the model toward assigning high probability to an event occurring around the observed time $t_{i}$ through the density term $f(t_{i}|z_{i};\theta )$ (equivalently, a log-density contribution $\log f(t_{i}|z_{i};\theta )$), whereas right-censored cases ($\delta_{i}=0$) pull the model toward assigning high probability of surviving beyond $t_{i}$ through $S(t_{i}|z_{i};\theta )$ (a log-survival contribution $\log S(t_{i}|z_{i};\theta )$). This formulation ensures that censored assets remain informative: the model is penalized if it predicts low survival probability up to the censoring time, which is essential in PdM where long fault-free operating histories are common. Learning-based survival models are therefore trained by maximizing L(θ) (or equivalently minimizing the negative log-likelihood −ℓ(θ)), which integrates both failures and censored observations within a single principled objective [72].

#### 4.5.1. Tree- and Ensemble-Based Models

Tree- and ensemble-based survival models extend classical non-parametric estimators by learning data-driven partitions of the covariate space and attaching a local survival estimate to each partition. The core idea is simple: assets with similar characteristics (e.g., age, design, environment, loading proxies) are grouped together through recursive splits, and each group is assigned its own empirical time-to-event behavior. Because censoring is handled explicitly during splitting and estimation, these methods can exploit both failures and long fault-free operating histories, making them particularly attractive as strong baselines in PdM settings [73].

An important non-parametric learning approach is the Random Survival Forest (RSF), which adapts random forests to survival data with censoring by growing many survival trees and averaging their predictions [74]. Each tree is trained on a bootstrap sample of the data. During tree construction, the algorithm repeatedly splits the dataset into two groups using a covariate and threshold that make the two groups as different as possible in terms of their survival experience (typically measured with log-rank-type criteria), while correctly handling censored observations.

After the tree is grown, each terminal node (leaf) *ℓ* contains assets that are similar in covariates and therefore expected to have similar failure behavior. The leaf then stores a simple empirical estimate of accumulated risk over time, usually the Nelson–Aalen cumulative hazard,

(37) $${\hat{H}}_{\ell }\left(t\right)=\sum_{t_{\left(j\right)}\leq t}\frac{e_{j,\ell }}{r_{j,\ell }}$$

where the sum runs over the event times $t_{\left(j\right)}$ observed among assets in that leaf, $e_{j,\ell }$ is the number of failures at time $t_{\left(j\right)}$, and $r_{j,\ell }$ is the number of assets still operating (at risk) just before $t_{\left(j\right)}$. In short, RSF groups similar assets and learns a local, censoring-aware risk curve for each group, then combines many such trees to obtain a stable survival prediction.

For a new asset with covariates *z*, each tree *m* routes *z* to a terminal node (leaf) and returns a leaf-level estimate of the cumulative hazard, denoted as ${\hat{H}}^{\left(m\right)}\left(t|z\right)$. A common forest-level aggregation averages these tree-specific cumulative hazards,

(38) $$\bar{H}\left(t|z\right)=\frac{1}{M}\sum_{m=1}^{M}{\hat{H}}^{\left(m\right)}\left(t|z\right)\;\bar{S}\left(t|z\right)=\exp\;\left(-\bar{H}\left(t|z\right)\right)$$

yielding a smooth and stable survival estimate $\bar{S}\left(t|z\right)$ for the asset. Equivalently, some implementations average the tree-level survival functions ${\hat{S}}^{\left(m\right)}\left(t|z\right)=\exp\left(-{\hat{H}}^{\left(m\right)}\left(t|z\right)\right)$ directly. In practical terms, RSF can be viewed as combining many weak survival estimators, each capturing a different partition of the feature space, into a robust, non-parametric predictor that models non-linear effects and high-order interactions, while remaining censoring-aware by construction.

From a PdM perspective, RSF is valuable because it (i) handles mixed numerical and categorical covariates with minimal preprocessing, (ii) naturally models interaction-driven risk mechanisms (e.g., soil type matters differently at different ages), and (iii) provides interpretable diagnostics such as variable importance and partial dependence-style summaries that support engineering validation. Related extensions include boosted survival trees and survival boosting variants, which can improve discrimination in some regimes at the expense of additional tuning complexity [57].

#### 4.5.2. Neural Cox (PH-Structured Deep Survival)

Neural Cox models preserve the proportional hazards (PH) structure of the classical Cox model, but replace the linear predictor $\beta^{⊤}z$ with a flexible, learned risk function $g_{\theta }\left(z\right)$. In practice, a neural network takes the covariates *z* as input and outputs a scalar log-risk score $g_{\theta }\left(z\right)$, allowing complex and non-linear combinations of features (e.g., interactions between operating conditions and asset attributes) to influence failure risk [75,76]. The hazard is modeled as

(39) $$h\left(t|z\right)=h_{0}\left(t\right)\exp\;\left(g_{\theta }\left(z\right)\right)$$

where $h_{0}\left(t\right)$ is an unspecified baseline hazard shared across the population, and $\exp(g_{\theta }\left(z\right))$ acts as an asset-specific relative-risk multiplier.

Training does not require specifying $h_{0}\left(t\right)$. Instead, the network parameters θ are learned by maximizing Cox’s partial likelihood, which compares each observed failure to the set of assets that were still at risk at that time. With the risk set $R\left(t_{i}\right)=\left\{j:\;t_{j}\geq t_{i}\right\}$, the standard objective is the negative log-partial-likelihood

(40) $$L_{\mathrm{Cox}}\left(\theta \right)=-\sum_{i:\;\delta_{i}=1}\left[g_{\theta }\left(z_{i}\right)\;-\;\log\;\sum_{j\in R(t_{i})}\exp\;\left(g_{\theta }\left(z_{j}\right)\right)\right]$$

Intuitively, Equation (40) encourages the model to assign higher risk scores to assets that fail earlier than their peers in the corresponding risk sets, while censored assets still contribute through their membership in $R(t_{i})$ whenever they remain under observation. Importantly, neural Cox models retain the Cox interpretability in terms of hazard ratios: for two assets with covariates $z_{1}$ and $z_{2}$,

(41) $$\frac{h(t|z_{1})}{h(t|z_{2})}=\exp\;\left(g_{\theta }\left(z_{1}\right)-g_{\theta }\left(z_{2}\right)\right)$$

so the model provides a principled relative ranking of failure risk while allowing non-linear covariate effects [77]. In PdM applications, neural Cox models are therefore attractive when proportional scaling of baseline risk is reasonable, but the covariate–risk relationship is too complex to be captured by a linear index, and censoring-aware training remains essential for learning from long fault-free operating histories.

Beyond the standard Cox model and its basic neural PH implementation, a broader set of Cox-oriented formulations has become important in modern survival modelling. In this review, these approaches are treated as extensions within the Cox-based semi-parametric and learning-based families rather than as separate top-level categories. Representative examples include DeepSurv-style models, which replace the linear risk term with a non-linear learned representation while retaining the proportional-hazards structure; penalized Cox variants, which improve robustness and variable selection in high-dimensional settings; multi-task Cox-type formulations, which enable shared representation learning across related asset groups, operating regimes, or failure mechanisms; and dynamic formulations that accommodate covariates updated over time. These extensions are particularly relevant in predictive maintenance because they preserve censoring-aware estimation and the familiar hazard-based interpretation of Cox models, while improving flexibility for heterogeneous sensor inputs, correlated features, and temporally evolving operating conditions.

#### 4.5.3. Neural Parametric/Neural Weibull Models

Neural parametric survival models take a direct probabilistic approach: instead of learning only a relative risk score (as in Cox-type models), they learn an asset-specific survival distribution. The key idea is to keep a familiar parametric lifetime family so that S(t∣z) and h(t∣z) remain explicit and operationally interpretable, but let a neural network predict the distribution parameters from covariates *z*. This combines the extrapolation and decision-friendly outputs of parametric reliability models (e.g., closed-form failure probabilities over a planning horizon) with the flexibility of deep learning to capture non-linear covariate effects and interactions [78].

A common and practically useful instance is the neural Weibull model, where the network outputs positive shape and scale parameters, α(z)>0 and λ(z)>0. The resulting survival and hazard functions are given in Equations (42) and (43), respectively:

(42) $$S\left(t|z\right)=\exp\;\left[-{\left(\frac{t}{\lambda (z)}\right)}^{\alpha (z)}\right]$$

(43) $$h\left(t|z\right)=\frac{\alpha (z)}{\lambda (z)}{\left(\frac{t}{\lambda (z)}\right)}^{\alpha (z)-1}$$

This parameterization has an intuitive interpretation: λ(z) sets the time scale of the lifetime (larger values imply longer expected life), while α(z) controls how failure intensity evolves with age (e.g., increasing wear-out when α(z)>1). By allowing both parameters to depend on *z*, the model can represent heterogeneous aging patterns across a fleet. For example, two assets with the same age may still have different hazard trajectories due to differences in environment, loading proxies, or condition indicators.

If α(z) is constrained to be constant (independent of *z*), the model reduces to a classical Weibull regression form where covariates mainly rescale lifetime through λ(z). In the fully flexible neural setting, both α(z) and λ(z) can vary with *z*, allowing covariates to change not only the time scale of failure but also the shape of the hazard curve (e.g., stronger wear-out acceleration for degraded assets). Training is performed by maximizing the censoring-aware likelihood introduced earlier, where failures contribute $\log f(t_{i}|z_{i})$ and censored cases contribute $\log S(t_{i}|z_{i})$ computed from the instance-specific Weibull. This yields an explicit survival curve per asset, enabling direct horizon risk calculations (e.g., Pr(T≤τ∣z)) and quantitative RUL estimation; when a single Weibull is insufficient, mixture-based extensions such as Deep Survival Machines can capture multi-modal lifetime patterns.

From an asset-management perspective, neural parametric models are attractive because they output a full survival curve per asset, enabling direct computation of planning quantities such as (i) failure probability within a horizon [0,τ], (ii) survival quantiles (e.g., median life), and (iii) time-dependent hazard-based prioritization. They also integrate naturally into simulation and optimization pipelines that require sampling or quantile evaluation. The main trade-off is that parametric assumptions matter: if the chosen family is too restrictive, the model may be miscalibrated, especially when extrapolating beyond the observed age range. To address multi-modal or highly heterogeneous lifetime patterns, mixture-based extensions (e.g., Deep Survival Machines) replace a single Weibull with a mixture of parametric components, while retaining a likelihood-based, censoring-aware training objective.

#### 4.5.4. Transformer and Sequence-to-Survival Models

Transformer and sequence-to-survival models represent the most expressive class of learning-based survival approaches, designed to exploit the high-dimensional and often temporally ordered data that arise in modern PdM settings. Unlike classical survival regression, which typically relies on a fixed set of handcrafted covariates, these models first learn a representation from heterogeneous inputs, such as mixed tabular features, event logs, or time-series sensor histories $x_{1:t}$, and then map that representation to a survival object (e.g., a risk score, a hazard function, or distribution parameters). The central benefit of attention-based architectures is that they can model complex cross-feature interactions and long-range temporal dependencies, which are common in degradation processes where risk is driven by combinations of operating regimes rather than any single variable.

A generic formulation is to encode the available information into a latent representation

(44) $$r_{\theta }={\mathrm{Transformer}}_{\theta }\left(\mathrm{tokens}\;\mathrm{or}\;x_{1:t}\right)$$

and then use a task-specific head to produce a survival prediction. For example, in a Cox-style (PH) design, the head outputs a log-risk score $g_{\theta }\left(z\right)$ and the hazard is written as Equation (39). Here, $g_{\theta }\left(z\right)$ is now computed from the transformer representation (and *z* may include static covariates together with features extracted from the sequence). Alternatively, the head can output parametric survival parameters (e.g., α(z) and λ(z) for a Weibull layer), yielding an explicit S(t∣z) as in neural parametric survival.

More broadly, transformer-based survival models are well suited when the available data are heterogeneous (categorical, numerical, and possibly sequential) and when degradation signatures depend on combinations of variables across time. However, these models require careful problem formulation to remain valid for survival prediction:

- **Leakage control:** the representation at time *t* must use only information available up to the censoring or prediction time; otherwise the model may learn post-failure patterns and overestimate performance.
- **Censoring-aligned sequences:** for right-censored units, the input history should terminate at the last observation time to avoid introducing artificial future context.
- **Calibration and evaluation:** highly expressive models can be overconfident, so calibration of predicted survival curves (and assessment with censoring-aware metrics) is often required before deployment in maintenance planning.

When these considerations are addressed, transformer and sequence-to-survival models provide a principled way to integrate rich sensor streams into survival-based RUL estimation and horizon risk forecasting, enabling more individualized and responsive PdM decisions.

Table 3 summarizes the corresponding formulas for parametric and learning-based survival models, including censoring-aware objectives used for model training.

### 4.6. Evaluation Metrics for Time-to-Event Prediction

Model evaluation for survival models must balance several aspects, including discrimination, calibration, accuracy of RUL estimates, and proper scoring. Key metrics in these categories and their interpretation for PdM are outlined.

#### 4.6.1. Discrimination (Ranking Ability)

Discriminative metrics evaluate how well a survival model can rank assets by their risk of earlier failure. A widely used measure is Harrell’s concordance index (C-index) [79]. The C-index is the proportion of usable asset pairs for which the model assigns higher risk to the asset that fails earlier. A C-index of 1.0 indicates perfect ranking, 0.5 corresponds to random ordering, and values below 0.5 indicate worse-than-random ranking performance [80]. In PdM, a high C-index implies the model reliably prioritizes high-risk assets (shorter RUL) ahead of low-risk assets for inspection or intervention. Extensions assess discrimination at specific horizons, such as time-dependent concordance measures that evaluate concordance up to a given time *t* or across time. Ref. [81] proposed an inverse probability of censoring weighting (IPCW)-adjusted C-index to improve performance under heavy censoring. Another common discrimination metric is the time-dependent AUC (area under the ROC curve), which treats survival prediction as a sequence of classification tasks at time *t*: distinguishing assets that fail by *t* from those that survive beyond *t* using a time-*t* risk score (often derived from S(t∣z)) [82]. For example, an AUC(t)=0.85 indicates that, at horizon *t*, a randomly chosen asset that fails by *t* is assigned higher risk than a randomly chosen asset that survives beyond *t* in 85% of such comparisons.

#### 4.6.2. Calibration

Calibration describes whether the model’s predicted risks can be trusted as actual probabilities. A simple intuition is that if 100 assets are each predicted to have a 20% chance of failing within the next month, then about 20 failures within that month should be observed [83]. Unlike discrimination metrics (which focus on ranking), calibration checks the numerical accuracy of predicted probabilities and survival curves, which is essential when predictions are used for planning and budgeting.

A common way to assess calibration is a calibration plot (reliability diagram). For a chosen horizon, assets are grouped by predicted risk (e.g., low to high predicted failure probability), and for each group the average predicted risk is compared with the observed fraction of failures. A well-calibrated model produces points close to the diagonal, meaning predicted equals observed. The expected calibration error (ECE) summarizes this idea with a single number: it averages the mismatch between predicted and observed frequencies across the groups (smaller is better). Since calibration can change with the forecast horizon, it is good practice to check more than one horizon; a model may appear calibrated for short-term risk but drift at longer horizons [81].

To evaluate calibration over the entire predicted time-to-event distribution (not just one horizon), distribution calibration (D-calibration) provides a more global check. In simple terms, it tests whether observed failures occur at times that are consistent with the model’s predicted survival curves across the population. Large systematic deviations indicate that the predicted distributions are misaligned with reality. In practice, calibration is often examined using a combination of visual diagnostics (e.g., comparing an average predicted survival curve with the Kaplan–Meier estimate) and summary statistics, to ensure that predicted risks are suitable for decision-making. In PdM, good calibration helps avoid unnecessary interventions caused by inflated risk estimates and missed failures caused by overly optimistic survival predictions [81].

#### 4.6.3. Accuracy of RUL Estimates

In PdM, it is often not enough to rank assets by risk; practitioners also need the predicted time to failure, usually reported as RUL. When a model outputs a single RUL value, a natural evaluation is the mean absolute error (MAE) between predicted and observed failure times: an MAE of 10 days means predictions are off by about 10 days on average. Under right censoring, however, the true failure time is unknown for many assets, so MAE cannot be computed directly for those cases [84]. Reporting MAE only on failed assets can therefore be biased, especially when long-lived assets are mostly censored. Common remedies include censoring-aware error definitions (e.g., using lower-bound errors for censored units) and estimators that incorporate censored data through pseudo-observations [85]. If uncertainty is reported (e.g., prediction intervals), coverage is also important: a nominal 90% interval should contain the true failure time about 90% of the time; deviations indicate over- or under-confidence. Overall, these measures complement discrimination by quantifying how accurate the predicted RUL is in practical time units.

#### 4.6.4. Proper Scoring Rules (Brier Score)

To evaluate survival predictions as probabilities (not only rankings), proper scoring rules compare the predicted survival curve to what actually happens. The most widely used is the Brier score (BS), which is a time-dependent analogue of mean squared error [86,87]. At a fixed horizon *t*, it measures how close the predicted survival probability $\hat{S}\left(t|x_{i}\right)$ is to the observed survival status by *t*. Specifically,

(45) $$\mathrm{BS}\left(t\right)=\frac{1}{n}\sum_{i=1}^{n}{\left(I\left\{T_{i}>t\right\}-\hat{S}\left(t|x_{i}\right)\right)}^{2}$$

where $I\{T_{i}>t\}$ equals 1 if the asset survives beyond *t* and 0 otherwise. Lower values indicate more accurate probabilistic predictions.

With right censoring, $I\{T_{i}>t\}$ is not always observable, so unbiased estimation typically uses IPCW, where each term is reweighted by the estimated chance of remaining uncensored up to *t* (often obtained from a Kaplan–Meier estimate of the censoring distribution). The Brier score is commonly reported as a function of time (a prediction error curve) and summarized by the Integrated Brier Score (IBS), which averages BS(t) over a time interval of interest [88]. Because the Brier score is a proper scoring rule, it rewards well-calibrated probabilities; unlike the C-index, it penalizes both misranking and miscalibration, making it particularly useful when PdM decisions depend on reliable horizon failure probabilities.

To make the evaluation criteria more explicit, several key metrics can be written in mathematical form. For discrimination at a specific horizon *t*, the time-dependent AUC is defined as

(46) $$\mathrm{AUC}\left(t\right)=\mathrm{Pr}\;\left({\hat{r}}_{i}\left(t\right)>{\hat{r}}_{j}\left(t\right)|T_{i}\leq t,\;T_{j}>t\right),$$

where ${\hat{r}}_{i}\left(t\right)$ denotes the predicted risk score for asset *i* at time *t*, and $T_{i}$ is the observed event time. This metric quantifies the probability that an asset failing by time *t* receives a higher predicted risk than one surviving beyond *t*.

For calibration at horizon *t*, let ${\hat{p}}_{i}\left(t\right)=1-\hat{S}\left(t|z_{i}\right)$ denote the predicted failure probability for asset *i*. If the assets are grouped into *B* bins, the expected calibration error (ECE) can be expressed as

(47) $$\mathrm{ECE}\left(t\right)=\sum_{b=1}^{B}\frac{n_{b}}{n}\left|{\bar{p}}_{b}\left(t\right)-{\bar{y}}_{b}\left(t\right)\right|,$$

where $n_{b}$ is the number of samples in bin *b*, *n* is the total number of samples, ${\bar{p}}_{b}\left(t\right)$ is the mean predicted failure probability in bin *b*, and ${\bar{y}}_{b}\left(t\right)$ is the corresponding observed failure frequency. Lower ECE values indicate better agreement between predicted and observed risk.

When the model outputs a point estimate of RUL, a basic error measure is the mean absolute error (MAE), given by

(48) $${\mathrm{MAE}}_{\mathrm{RUL}}=\frac{1}{n_{f}}\sum_{i:\delta_{i}=1}\left|{\hat{\mathrm{RUL}}}_{i}-{\mathrm{RUL}}_{i}\right|,$$

where ${\hat{\mathrm{RUL}}}_{i}$ is the predicted RUL for asset *i*, ${\mathrm{RUL}}_{i}$ is the true remaining useful life, $\delta_{i}$ is the event indicator, and $n_{f}$ is the number of observed failures.

Finally, the IBS over the interval [0,τ] is defined as

(49) $$\mathrm{IBS}\left(\tau \right)=\frac{1}{\tau }\int_{0}^{\tau }\mathrm{BS}\left(t\right)\;dt,$$

where BS(t) is the Brier score at time *t*. The IBS summarizes the overall probabilistic prediction error across time, with lower values indicating better predictive performance.

## 5. Applications of Survival Models in Energy Systems

Electricity DSOs and DHN companies increasingly embed survival analysis into asset management to translate predictive insights into operational decisions. DSOs can rank assets by failure risk and schedule replacements before end-of-life, and DHNs can plan proactive pipe repairs or intensified monitoring on vulnerable segments. In the following, practical applications in electricity distribution and district heating asset management are discussed.

### 5.1. Electricity Distribution Applications

Survival analysis has been applied to a range of electricity distribution assets, often grouped by asset class.

#### 5.1.1. Underground Cables

MV and low-voltage (LV) power cables are a major focus due to their extensive installed length and failure impact. Cox proportional hazards models are frequently used to identify which cable attributes drive failure risk. For example, ref. [89] analyzed historical failure data for MV distribution cables (10 kV) and high-voltage (HV) transmission cables (110–220 kV) from a Chinese utility using a multivariate Cox PH model, finding that certain covariates (like cable type and joint manufacturer) increase hazard rates. This Cox approach proved more robust than a homogeneous Weibull fit for these cables, as it could accommodate heterogeneous populations and even single out a problematic batch of cable joints (a manufacturing defect) for targeted replacement.

**Figure 6:**
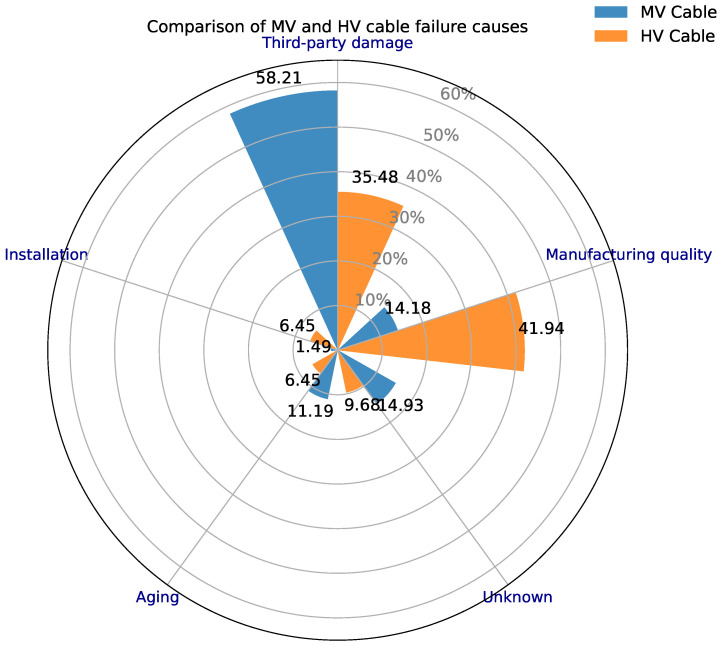
Failure-cause composition of MV and HV cable populations based on [89].

Figure 6 highlights a clear shift in the dominant failure mechanisms between MV and HV cable populations [89]. MV cable failures are primarily associated with third-party damage, whereas HV failures are more strongly linked to manufacturing-quality-related causes, with smaller contributions from unknown, aging, and installation-related factors. This contrast underscores the heterogeneous nature of cable deterioration and reinforces the need for covariate-aware survival models rather than homogeneous lifetime assumptions.

Similarly, ref. [48] applied survival modeling to LV cable networks, quantifying how factors such as conductor material, loading level, and soil conditions affect cable failure frequencies. Their study demonstrated that incorporating such covariates enables more refined asset health indices, improving the asset manager’s ability to prioritize cable sections for intervention. Other studies have extended cable failure analysis, such as [90], which performed spatial survival analysis on aging paper-insulated cables to pinpoint regions of accelerated deterioration. Ref. [3] reported baseline failure rates for MV XLPE cables from national statistics, providing a benchmark for probabilistic life models. Ref. [91] compared Weibull and Crow-AMSAA models for early cable joint failures, finding that a Weibull-based hazard model better captured the increasing failure rate after an initial infant mortality period. In recent years, machine learning (ML) approaches have also been applied to cable maintenance.

**Figure 7:**
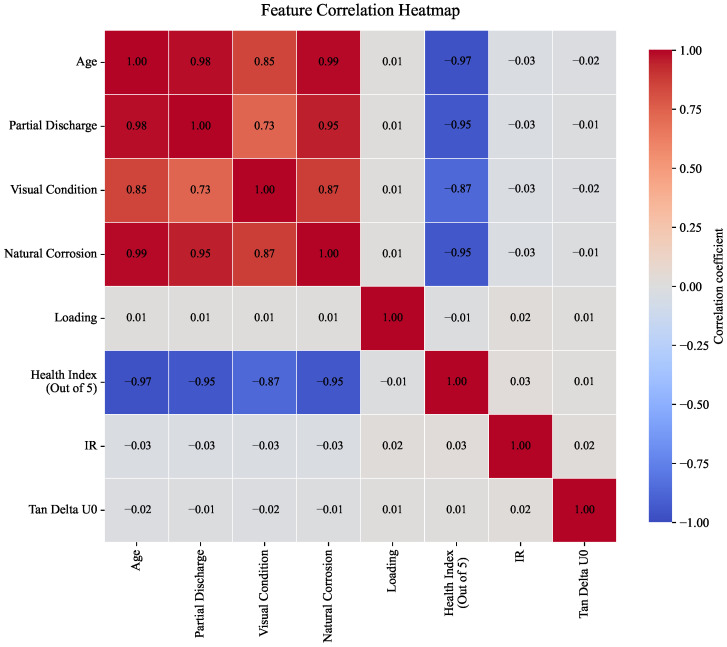
Correlation heatmap of cable condition variables and health index for in-service XLPE cables, adapted from [92].

Ref. [92] developed an artificial neural network model to predict the insulation health index of in-service XLPE cables, allowing early identification of degrading cable segments and thus supporting proactive replacements. Across these studies, cable failure models support renewal planning by identifying high-risk cables (beyond what age alone would indicate) and by estimating the residual life benefits of mitigations like improved installation practices. In addition, the correlation analysis reported in [92] shows that cable age is strongly associated with key degradation indicators, particularly partial discharge and natural corrosion, while the health index exhibits a pronounced inverse relationship with these variables (Figure 7). This further highlights that cable deterioration is shaped by interacting electrical, environmental, and condition-related factors rather than by age alone, thereby reinforcing the value of multivariate, data-driven models for cable condition assessment and maintenance decision support.

Overhead distribution lines have also been studied with survival analysis techniques, although failures are rarer in lines than in aging cables. An illustrative example is [93], which developed a hierarchical Bayesian model for overhead line failure rates. They combined outage data from multiple regions and line populations, accounting for population variability (differences in line design, environment, and maintenance history) through random effects in a Poisson regression model. This approach (a Bayesian survival regression) allowed them to estimate credible failure rate distributions for overhead lines even with sparse failure observations. By including region-specific random effects, the model identified which line sub-populations had higher baseline hazard.

#### 5.1.2. Distribution Transformers

Aging distribution transformers (typically 10–20 kV) have also been studied with survival models to support maintenance and replacement decisions. A common approach is to fit parametric lifetime distributions or Cox regressions to long-term failure data. Ref. [94] investigated failure and retirement statistics for a fleet of Australian power transformers spanning multiple voltage levels, using non-parametric survival curves (Kaplan–Meier estimates) and parametric fits (Weibull models) to characterize the transformers’ lifetimes. The fitted Weibull models allowed them to estimate age-dependent failure probabilities and thus suggest optimal replacement ages for different classes of transformers. Ref. [95] took a two-part approach for utility transformers: first modeling aging-related failures with a Weibull time-to-failure law, then extending to a combined model with both random (chance) failures and wear-out failures. By using Monte Carlo simulations based on these Weibull hazard models, they showed how many years of data (and how many failure events) are needed to confidently predict transformer end-of-life which is an important practical insight for utilities with limited failure records. More recently, attention has turned to time-varying covariates that influence transformer survival. Ref. [96] integrated eleven years of distribution transformer failure data from South Korea with detailed local weather records in a Cox PH model to quantify how environmental stress (e.g., high ambient temperature and humidity) contributes to transformer failures. They report that extreme hot and humid conditions correspond to elevated hazard ratios for transformer failure. In a similar vein, the RUL of distribution transformers under rising electric vehicle (EV) loading stress is estimated by [19], finding that heavy EV charging penetration measurably accelerates the transformers’ wear-out rate. To incorporate multiple degradation processes, some works combine physics-based and data-driven models. For example, ref. [97] proposed a hybrid prognostics framework for power transformers that blends a physical degradation model with survival analysis, improving failure time predictions under fluctuating renewable loading conditions.

#### 5.1.3. Switchgear and Substation Devices

Beyond cables and transformers, utilities have applied survival analysis to switchgear components such as disconnectors, circuit breakers, and instrument transformers. Ref. [98] examined MV disconnect switches and reclosers using a Cox model, leveraging failure logs before and after certain interventions. Specifically, the installation of remote control and the adoption of preventive overhaul to compare hazard rates. The Cox analysis showed that remotely controlled switches had a lower hazard of failure (since manual switching operations were eliminated), and that devices receiving periodic maintenance likewise exhibited reduced failure rates. This translates into decision support for maintenance policy. The model provided quantitative evidence that automation and proactive overhaul both extend the survival of switchgear, thereby averting in-service failures. Ref. [99] analyzed the reliability of high-voltage instrument transformers (voltage and current transformers) using survival methods. They employed Kaplan–Meier estimators to establish baseline failure probabilities over time and Cox regression to assess covariate effects such as loading, manufacturer, and installation environment. The resulting failure rate estimates enabled a risk-ranked replacement plan for the utility. Instrument transformers with the highest estimated risk (for example, certain models operating under high loading stress) were prioritized for pre-emptive replacement, thereby preventing potential outages. In the realm of circuit breakers, statistical life models have also been applied. For instance, ref. [100] analyzed field failure records of HV SF_6_ breakers and fitted a Weibull distribution to determine the wear-out age of breaker contacts. This study provided an estimated life distribution for breakers and recommended overhaul intervals (e.g., mid-life refurbishments) to prevent in-service failures. Additionally, some studies adopt Bayesian or data-driven updating approaches using condition-monitoring data (e.g., contact wear measurements or the number of operations) to continually refine breaker failure rate estimates. In all these cases, survival-based methods convert historical performance and condition data into actionable insights, identifying which substation components are most likely to fail next and what operational measures can mitigate that risk.

### 5.2. District Heating Network Applications

Statistical life modeling has only recently been applied to DHN systems, which historically relied on deterministic life estimates. Ref. [101] outline a new asset management approach for DHNs that replaces static lifespan assumptions with data-driven failure predictions, explicitly suggesting the use of survival analysis to prioritize pipe renewals based on observed failure trends. Following this vision, authors in [13] conducted one of the first comprehensive survival analyses on a modern DHN pipe network. They gathered about 20 years of failure records for underground steel pipe segments in a Danish district heating system, alongside each pipe’s characteristics (age, diameter, insulation type, soil type) and operating history. Given that the network is relatively young (many pipes had not yet failed), the authors tested both classical and novel survival models while addressing the data scarcity. In particular, they compared a Weibull proportional hazards model (a parametric cohort model often used in water distribution pipes, derived from Herz’s exponential wear-out model) and a modern neural-network-based Cox model (neural Weibull PH) that can flexibly fit hazard functions even with missing data. To accommodate common data deficiencies (such as unknown installation dates or censored failure times), they modified the likelihood functions of these models which allowed the survival analysis to make the best use of incomplete records. The outcome of this study was two-fold; first, they found that data-driven models can indeed differentiate pipe segments by risk better than the traditional age-based approach. For example, pipes in certain corrosive soil types or with higher pressure cycling showed measurably higher hazards, even if not the oldest. Second, their evaluation highlighted the difficulty of validating distributional assumptions in young networks. However, the Weibull model’s wear-out shape could not be conclusively confirmed due to the limited number of late-life failures, suggesting that pooling data from multiple utilities or waiting for more failure observations is necessary to refine long-term life estimates. For the DHN operator, the practical output was a ranked list of pipe sections by failure probability, enabling a shift from purely reactive maintenance to prioritized renewal of the highest-risk pipes.

Reference [102] performed an earlier reliability analysis on a district heating system, developing an integrated failure probability assessment for a municipal DHN network. This work underscored the need for more sophisticated statistical models (like survival analysis) to supplement basic lifetime estimates in DHN infrastructure. Beyond purely data-driven models, other DHN pipe studies have drawn on system reliability methods as well. Ref. [103] approached the problem from a network reliability perspective, simulating a repairable DHN system under varying external conditions to evaluate its availability and failure frequency. By modeling how seasonal demand swings and temperature fluctuations affect pipe stress and leak rates, they provided insight into how the hazard of pipe failure might increase during peak load periods (late winter, when pipes have endured maximum thermal stress), which is useful for maintenance scheduling. Although such system-level analyses are not survival models in a strict sense, they complement component-level survival predictions by examining the operational contexts in which those failures occur.

Beyond pipelines, some work has applied survival analysis to district heating equipment like pumps and valves, though this area is less developed. Ref. [13] notes in their PdM survey of DHN that mechanical components (e.g., circulation pumps in boiler houses) could also benefit from survival-based failure modeling, despite the limited run-to-failure data available for such equipment. For instance, ref. [104] developed a Random Survival Forest model for large water pumps, which was capable of handling small samples of failure data by leveraging ensemble learning. The model identified an early infant mortality phase in pump failure rates followed by a longer period of lower hazard, aligning with the typical bathtub curve, and used this insight to optimize maintenance intervals for pumps (i.e., more frequent checks early on, then steady monitoring during the long reliable period). By analogy, DHN operators can apply similar approaches to predict failures of critical pumps in heat production plants or substations, improving planning for spare units and overhauls [105]. Overall, however, pipes remain the primary focus in DHNs, and as more failure data accumulate, increasing use of survival models (from simple Weibull analyses to advanced ML survival models) is expected to guide pipe replacement planning, leak detection strategies, and life-extension measures (like cathodic protection retrofits and re-insulation scheduling).

**Figure 8:**
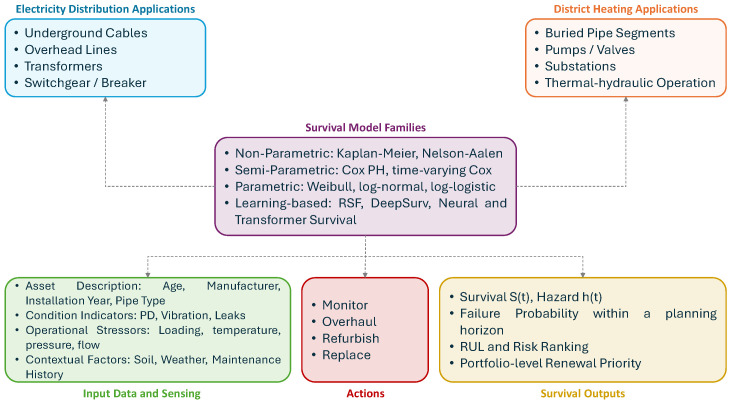
Overview of survival-model applications, inputs, outputs, and maintenance actions across electricity distribution and district heating systems.

Figure 8 summarizes the application landscape discussed in this section by linking representative asset classes in electricity distribution and district heating to the main survival-model families, typical input data, and decision-oriented outputs. As illustrated, these applications rely on combining asset descriptors, condition indicators, operational stressors, and contextual information to estimate survival, hazard, failure probability, and risk ranking. This integrated view highlights that survival analysis in smart energy networks is not only a modelling framework for failure prediction, but also a practical decision-support tool for inspection planning, maintenance prioritization, refurbishment, and replacement.

### 5.3. Public Benchmark Datasets and Reproducibility

Publicly available benchmark datasets for predictive maintenance and time-to-event modelling in smart energy systems remain limited, especially for asset classes with explicit failure times, censoring information, and continuous asset-level histories. To support reproducible evaluation and practical adoption, Table 4 summarizes representative public datasets in terms of asset type, data modalities, time resolution, sample size, and whether failure times, censoring, or delayed entry can be constructed.

### 5.4. Practical Model Selection

The selection of a survival modeling approach in practice is closely tied to (i) asset observability (what data are available and whether they are static or time-varying), (ii) fleet heterogeneity, and (iii) the type of maintenance decision to be supported. If only static asset data (e.g., installation age and design type) and sparse failure events are available, utilities tend to favor simpler, interpretable models. For instance, a Weibull lifetime analysis or a Cox PH model with static covariates is often sufficient to rank assets by risk (addressing point (i)).

On the other hand, when rich condition-monitoring data or time-series sensor inputs exist (vibrations, load profiles, thermal measurements), more dynamic models become viable, such as extended Cox models that allow time-dependent covariates, or even sequence-based survival models, can exploit this greater observability to update failure risk in real time [114].

Regarding fleet heterogeneity, a very diverse asset population (in terms of ages, manufacturers, operating environments) might require models that capture unit-specific random effects or that partition the fleet. For example, a stratified Cox model or a Random Survival Forest can accommodate different sub-populations without forcing one-size-fits-all parameters. Conversely, a homogeneous fleet (assets of similar type and age) can often be effectively modeled with a single parametric distribution, assuming that after accounting for the available covariates, each unit’s failure time follows the same distribution and is independent of the others.

Finally, the decision context (point (iii)) drives whether the model should output absolute failure time predictions or just relative risk rankings. For risk ranking of assets (choosing which components to inspect or replace first), models like the Cox PH (which excel at relative hazard estimation) are well suited and widely used. However, for time-based planning, engineers often need the full time-to-failure distribution or an expected remaining life. In those cases, parametric survival models or other approaches that yield an explicit survival function (possibly calibrated to real failure probabilities) are preferred, as they can answer “how long until failure?” in probabilistic terms, directly informing maintenance timelines.

The reviewed model families differ not only in predictive flexibility but also in the type and quality of data they require for reliable use. Non-parametric methods such as Kaplan–Meier and Nelson–Aalen are most appropriate when the goal is descriptive fleet-level reliability estimation under censoring, but they are limited when individualized prediction is needed or when many covariates must be incorporated. Parametric models such as Weibull, exponential, or log-logistic are attractive when the available dataset is relatively small, the number of observed failures is limited, and the failure process can be approximated by a simple hazard shape; however, their performance can degrade when the assumed lifetime distribution is poorly matched to the asset physics or operating environment. Semi-parametric models such as Cox PH are often a practical compromise, but they still require sufficiently informative covariates, a reasonable number of events, and at least approximate compatibility with proportional-hazards behaviour if stable interpretation is desired. When time-varying covariates are used in Cox-type models, the data must also support reliable temporal alignment between sensor updates, asset states, and event times.

Tree-based and ensemble methods such as random survival forests are generally more suitable when larger datasets are available and when the relationships between condition indicators and failure risk are non-linear or involve interactions that are difficult to specify manually. However, these models can become unstable when event counts are low, censoring is heavy, or the signal-to-noise ratio of the covariates is weak, because the recursive splits may then reflect sampling noise rather than persistent degradation structure. Learning-based survival models, including deep and Transformer-based approaches, typically impose the strongest data requirements. In practice, they benefit from larger cohorts, richer longitudinal sensing, consistent feature definitions across assets, and careful preprocessing of missing values, irregular sampling, and label uncertainty. Without such data support, these models may overfit, learn spurious operational patterns, or produce predictions that are difficult to validate and interpret in engineering settings. Therefore, the suitability of a survival model should be judged not only by its modelling capacity, but also by whether the available dataset provides adequate event frequency, censoring structure, temporal consistency, and covariate quality for that model family.

In sum, data availability, model interpretability, and decision objectives must be balanced by practitioners; a simple Cox or Weibull model may suffice to prioritize assets by risk, whereas more complex or data-hungry models are justified when high-frequency condition data are available or when precise estimates of failure timing are needed.

From an engineering perspective, the choice between learning-based and classical survival models involves a trade-off between flexibility and deployability. Learning-based models such as RSF and Transformer-based survival models can better capture non-linear interactions and heterogeneous condition patterns, but they often require larger datasets, higher computational effort, and offer less transparent decision logic. These limitations can be important in utility applications, where failures are sparse, censoring is substantial, and model outputs must remain auditable for engineering decision-making. In contrast, parametric and semi-parametric models such as Weibull and Cox PH remain attractive in small- to medium-sample settings because they train quickly, impose low computational burden, and provide interpretable parameters or hazard ratios. Although their structural assumptions may limit flexibility, they often provide a more robust and practical starting point for engineering deployment.

For practical deployment, survival-based PdM can be structured as an end-to-end workflow. First, raw SCADA, AMI, or sensor measurements are collected, aligned to asset identifiers and timestamps, and aggregated into decision-relevant summaries. Next, the event of interest and censoring status are defined for each asset, followed by the construction of static and time-varying covariates while preventing information leakage. The selected survival model is then trained using censoring-aware estimation and evaluated in terms of discrimination, calibration, and probabilistic accuracy. Based on the estimated survival function, horizon-specific failure risk and RUL summaries can be derived for each asset. These outputs are finally translated into maintenance actions, such as inspection, replacement, or prioritization, and the workflow is updated periodically as new operational data becomes available.

## 6. Practical Challenges in PdM Survival Modelling

### 6.1. Data Availability, Quality, and Covariate Integration

An important practical point is that model performance depends not only on the choice of survival method but also on the representativeness and preparation of the input data. Many published studies report results on well-curated and already validated datasets, where labels, observation windows, and failure definitions are relatively clean. In real utility settings, however, sensor data are often noisier, incomplete, weakly aligned with asset events, and affected by changing operating practices. Under such conditions, methods that perform well on curated benchmark data may become unstable or show limited practical value unless preprocessing, event definition, missing-data handling, and leakage control are addressed carefully. Therefore, reliable RUL and PdM analysis requires representative data preparation as a first-order engineering task rather than only a model-selection step.

Despite the diversity of assets, several common themes emerge across the reviewed applications in power and heat networks. Data availability and quality is a universal concern. Utilities often have sparse failure data (since failures are infrequent by design) and many censored observations. For example, performing survival analysis on a relatively young DHN system runs the risk of not observing the wear-out phase at all, making it hard to validate the assumed failure distribution. Similarly, early reliability studies of water and power networks stress that richer failure datasets (spanning a wide range of asset ages, types, and environments) greatly improve model confidence [93]. This has led to an emphasis on integrating heterogeneous data sources to enhance predictive power. Many studies combine failure logs with other data: asset GIS records (installation dates, asset types, locations), operational sensor readings (loads, temperatures, pressures), environmental factors (soil corrosivity for pipes, weather for overhead lines), and even maintenance history. The inclusion of covariates from these sources is evident in numerous works, from weather effects in transformer models [96] to diagnostic test results in cable models (e.g., partial discharge or insulation health index measurements are sometimes used to refine cable hazard estimates [9,115]. A practical pattern is that adding such covariates can improve prediction accuracy if sufficient historical data exist to reliably estimate their effects. Otherwise, there is a risk of overfitting or inconclusive results. Data limitations have also spurred methodological adaptations, such as Bayesian updating techniques and modified likelihood functions, to make the most of small samples and incomplete information. For example, a recent study combined Bayesian network modeling with Weibull analysis to incorporate expert knowledge and conditional dependencies in cable failure predictions [116]. In general, utilities have adopted stopgap measures like expert elicitation and conservative design margins to cope with sparse data, but the trend is toward systematically augmenting historical data with new sensor streams and multi-utility data pooling to improve survival model robustness.

### 6.2. Model Selection, Complexity, and Interpretability

Another theme is the choice of survival model family in relation to the use-case requirements. Simpler models (Kaplan–Meier curves, exponential/Weibull parametric distributions, Cox PH with static covariates) remain popular in operational studies because of their interpretability and relatively low data demands. For instance, the Cox model’s regression coefficients offer clear insight into which asset factors are risk-increasing or risk-mitigating, which is valuable for engineering judgment and has been exploited in multiple asset classes. Likewise, parametric models like the Weibull model are appreciated for yielding an explicit remaining-life distribution that can directly answer the question “how many years until failure with X% probability?,” aligning well with maintenance scheduling needs. On the other hand, more complex models (ensemble and ML approaches) are gradually appearing as asset datasets grow in size and detail. Random Survival Forests and neural-network–based survival models can capture non-linear covariate interactions and even time-dependent effects, as seen in recent pump and cable case studies [92,104]. These advanced models often achieve higher predictive performance, especially when there is a wealth of sensor data or high-dimensional inputs, but they introduce practical challenges; the results can be harder to interpret for asset managers, and they typically demand larger datasets for training. A recurrent point across the literature is the need to balance model complexity with transparency. In critical infrastructure contexts, maintainers must trust and understand the model’s recommendations. Thus, there is often a preference for models that provide explainable rankings or factor effects, unless the expected operational value strongly justifies a black-box approach. In such cases, post hoc interpretability tools (e.g., feature attribution and rule-based explanations) can be used to improve transparency and facilitate adoption [117].

### 6.3. Decision Support and Operational Use

A third common pattern is how the outputs of survival models are used for decision support. Virtually all the application studies tie the statistical predictions back to maintenance or renewal actions. A predominant use is risk-based asset ranking, where models estimate either the hazard rate or the probability of failure within a given horizon to produce a prioritized list of components. In a broader sense, survival models enable a shift from reactive or calendar-based maintenance to predictive prioritization. Rather than treating all assets as equally likely to fail, resources can be focused on the statistically most vulnerable assets. Another operational output is an estimate of expected lifetime or time-to-failure for asset populations, which is crucial for long-term renewal planning. By fitting a survival curve to asset failure data, utilities can forecast how many failures to expect in the next 5, 10, or 20 years if no action is taken. This informs budgeting and resource allocation. Similarly, other authors have used survival-derived failure projections to compare alternative replacement strategies or to justify the costs of preventive maintenance programs in terms of avoided failures [118]. Across both electricity and heating domains, the regulatory context (such as reliability-centered regulations or performance-based incentives) is also seen to be pushing operators to adopt these quantitative risk models. For instance, power distributors under performance-based regulation use survival model outputs to demonstrate that their targeted replacement of high-risk cables will improve reliability indices (SAIDI/SAIFI) by a calculable amount. In district heating, where reducing leak rates and improving energy efficiency are growing concerns, survival analysis helps quantify the benefit of proactive pipeline renewals in terms of avoided water loss and heat supply disruptions. Finally, it is noteworthy that the power and DHN sectors are learning from parallel work in the water supply industry. Decades of pipe break modeling research (using statistical survival and reliability models) provide methodologies that can be transferred to energy pipelines [119,120]. Concepts like cohort survival models for aging infrastructure and the importance of combining expert knowledge with data-driven models are cross-cutting lessons increasingly influencing how survival analysis is applied in smart energy networks.

## 7. Conclusions

Sensor-enabled smart energy networks increasingly rely on data-driven reliability management to reduce unplanned outages and support risk-informed maintenance planning. In electricity distribution and district heating networks, time-to-event datasets are shaped by the realities of field operation: observation windows are limited, preventive interventions terminate follow-up, and only a fraction of assets fail during the study period. As a result, censoring and truncation are not peripheral details but defining characteristics of the modeling problem, and they must be handled explicitly in both model formulation and evaluation. This review organized survival modeling approaches for PdM and RUL estimation into a coherent taxonomy spanning non-parametric, semi-parametric, parametric, and learning-based families. Across these model classes, how assumptions interact with the structure of available data was highlighted, ranging from static asset metadata to time-varying covariates derived from sensors, inspections, and operational context. Beyond model choice, these families were mapped to the forms of decision support typically required in practice, including risk ranking, failure probabilities over planning horizons, and full predictive distributions over remaining lifetime. A consistent message is that progress depends as much on disciplined validation as on model sophistication. Reliable PdM and RUL pipelines require evaluation protocols that respect censoring/truncation and that measure both ranking quality and probabilistic accuracy. For researchers, the taxonomy in this paper provides a structured basis for positioning new methods, making assumptions explicit, and reporting results in a manner that is comparable and reproducible. For practitioners, the mapping from model families to data requirements and decision outputs provides a pragmatic guide for selecting and deploying survival models that align with operational objectives and available instrumentation.

## Figures and Tables

**Table 1:** Comparison between this review and related state-of-the-art review publications.

Reference	Application/Asset	Cen	NonPar	SemiPar	Par	Learn	Key Emphasis
[22]	General PHM (RUL)	**✗**	**✗**	**✗**	**✗**	✓	GNN RUL
[23]	General PHM (RUL)	**✗**	(✓)	(✓)	✓	✓	PI RUL
[24]	General PHM (RUL)	**✗**	**✗**	(✓)	(✓)	✓	DL RUL
[25]	Cross-domain	✓	(✓)	✓	✓	✓	DL survival
[26]	General PdM	(✓)	**✗**	(✓)	(✓)	✓	PdM survey
[27]	Rotating machinery	**✗**	**✗**	**✗**	(✓)	(✓)	RotMach PdM
[28]	General assets	**✗**	(✓)	**✗**	(✓)	✓	AD+RUL
[29]	Solar PV systems	**✗**	**✗**	**✗**	**✗**	(✓)	PV reliability
[30]	Power transformers	**✗**	**✗**	**✗**	**✗**	**✗**	AI+IoT
[31]	Wind turbines	**✗**	**✗**	**✗**	**✗**	(✓)	PdM+PrsM
[32]	Renewable energy	**✗**	**✗**	**✗**	**✗**	✓	RES optimization
[33]	General equipment	**✗**	**✗**	(✓)	(✓)	✓	AI RUL
This paper	Smart energy networks	✓	✓	✓	✓	✓	Survival taxonomy

*Legend*: ✓ = explicitly covered; (✓) = briefly/partially mentioned; **✗** = not covered. *Abbreviations*: Cen = censoring/truncation; NonPar = non-parametric; SemiPar = semi-parametric; Par = parametric; Learn = learning-based; PHM = prognostics and health management; AD = anomaly detection; AI = artificial intelligence; DL = deep learning; GNN = graph neural network; IoT = internet of things; PI = prediction interval; PrsM = prescriptive maintenance; PV = photovoltaic; RES = renewable energy sources; RotMach = rotating machinery.

**Table 2:** Summary of core survival-model formulas (non-/semi-parametric).

Model/Quantity	Formula(s)	Notes
Core functions	S(t)=Pr(T>t) $h\left(t\right)=\lim_{\Delta t\to 0}\frac{\mathrm{Pr}(t\leq T<t+\Delta t|T\geq t)}{\Delta t}$ $H\left(t\right)=\int_{0}^{t}h\left(u\right)\;du,\;S\left(t\right)=\exp\left[-H\left(t\right)\right]$	Survival, hazard, cumulative hazard
Kaplan–Meier (KM)	$\hat{S}\left(t\right)=\prod_{t_{\left(j\right)}\leq t}\left(1-\frac{e_{j}}{r_{j}}\right)$	Non-parametric S(t)
Nelson–Aalen (NA)	$\hat{H}\left(t\right)=\sum_{t_{\left(j\right)}\leq t}\frac{e_{j}}{r_{j}}$ $\hat{S}\left(t\right)=\exp\left[-\hat{H}\left(t\right)\right]$	Non-parametric H(t) and implied S(t)
Cox PH (proportional hazard)	$h\left(t|z\right)=h_{0}\left(t\right)\exp\left(\beta^{⊤}z\right)$	Semi-parametric; unspecified $h_{0}\left(t\right)$
Cox (extended; time-varying)	$h\left(t|z\left(t\right)\right)=h_{0}\left(t\right)\exp\;\left(\sum_{j=1}^{p_{1}}\delta_{j}z_{j}\left(t\right)+\sum_{i=1}^{p_{2}}\beta_{i}z_{i}\right)$	Time-varying and static covariates
Cox partial likelihood	$L\left(\beta \right)=\prod_{i:\;\delta_{i}=1}\frac{\exp(\beta^{⊤}z_{i})}{\sum_{j\in R(t_{i})}\exp\left(\beta^{⊤}z_{j}\right)}$	$R\left(t_{i}\right)=\left\{j:t_{j}\geq t_{i}\right\}$

**Table 3:** Summary of core survival-model formulas (parametric and learning-based).

Model/Quantity	Formula(s)	Notes
Weibull	$h\left(t\right)=\frac{\alpha }{\lambda }{\left(\frac{t}{\lambda }\right)}^{\alpha -1}$ $S\left(t\right)=\exp\;\left[-{\left(\frac{t}{\lambda }\right)}^{\alpha }\right]$	Parametric; monotone hazard
Exponential	$h\left(t\right)=\frac{1}{\lambda }$ $S\left(t\right)=\exp\;\left(-\frac{t}{\lambda }\right)$	Weibull with α=1
Gompertz	$h\left(t\right)=b\;e^{at}$ $S\left(t\right)=\exp\;\left[-\frac{b}{a}\left(e^{at}-1\right)\right],\;a\neq 0$	Exponentially changing hazard
Log-normal (AFT)	$\ln T=\beta^{⊤}z+\sigma \epsilon ,\;\epsilon ∼N\left(0,1\right)$ $S\left(t\right)=1-\Phi \;\left(\frac{\ln t-\mu }{\sigma }\right)$	Non-monotone hazard possible
Log-logistic	$S\left(t\right)=\frac{1}{1+{\left(\lambda t\right)}^{k}}$ $h\left(t\right)=\frac{\lambda k\;t^{k-1}}{1+{\left(\lambda t\right)}^{k}}$	Peaked hazard when k>1
Gamma	$f\left(t\right)=\frac{1}{\Gamma \left(\kappa \right)\theta^{\kappa }}t^{\kappa -1}\exp\;\left(-\frac{t}{\theta }\right)$ $S\left(t\right)=\frac{\Gamma (\kappa ,t/\theta )}{\Gamma (\kappa )}$, $h\left(t\right)=\frac{f(t)}{S(t)}$	Uses (in)complete Gamma functions
Censoring-aware likelihood	$L\left(\theta \right)=\prod_{i=1}^{n}f{\left(t_{i}|z_{i};\theta \right)}^{\delta_{i}}\;S{\left(t_{i}|z_{i};\theta \right)}^{1-\delta_{i}}$ $\ell \left(\theta \right)=\sum_{i=1}^{n}\left[\delta_{i}\log f\left(t_{i}|z_{i};\theta \right)+\left(1-\delta_{i}\right)\log S\left(t_{i}|z_{i};\theta \right)\right]$	Likelihood-based training
RSF (leaf NA)	${\hat{H}}_{\ell }\left(t\right)=\sum_{t_{\left(j\right)}\leq t}\frac{e_{j,\ell }}{r_{j,\ell }}$	Leaf cumulative hazard
RSF aggregation	$\bar{H}\left(t|z\right)=\frac{1}{M}\sum_{m=1}^{M}{\hat{H}}^{\left(m\right)}\left(t|z\right)$ $\bar{S}\left(t|z\right)=\exp\;\left(-\bar{H}\left(t|z\right)\right)$	Forest prediction
Neural Cox	$h\left(t|z\right)=h_{0}\left(t\right)\exp\;\left(g_{\theta }\left(z\right)\right)$ $L_{\mathrm{Cox}}\left(\theta \right)=-\sum_{i:\;\delta_{i}=1}\left[g_{\theta }\left(z_{i}\right)-\log\;\sum_{j\in R(t_{i})}\exp\;\left(g_{\theta }\left(z_{j}\right)\right)\right]$	PH-structured deep survival
Neural Weibull	$S\left(t|z\right)=\exp\;\left[-{\left(\frac{t}{\lambda (z)}\right)}^{\alpha (z)}\right]$ $h\left(t|z\right)=\frac{\alpha (z)}{\lambda (z)}{\left(\frac{t}{\lambda (z)}\right)}^{\alpha (z)-1}$	Neural network outputs α(z),λ(z)
Transformer representation	$r_{\theta }={\mathrm{Transformer}}_{\theta }\left(\mathrm{tokens}\;\mathrm{or}\;x_{1:t}\right)$	Sequence encoder

**Table 4:** Representative public datasets relevant to predictive maintenance and time-to-event modelling in smart energy systems.

Asset Type	Data Modalities	Time Resolution	Sample Size
District heating substations [106]	Operational time series and fault/event records	10-min	93 substations; 73 faults
Wind turbines [107,108]	SCADA measurements and fault labels	10-min	36 turbines; 95 datasets
Overhead power lines [109]	Three-phase voltage signals with fault labels	40 MHz	2904 measurements
Electric-machine insulation/partial discharge [110]	Multi-channel acoustic recordings	96 kHz	12,850 samples
Power transformers [111]	Dissolved gas analysis measurements with fault labels	Tabular	376 samples
Power transformers [112]	DGA measurements with diagnosis labels	Tabular	589 samples
Power transmission lines [113]	Simulated voltage/current features with fault labels	Tabular	618 samples

## Data Availability

No new data were created or analyzed in this study. Data sharing is not applicable to this article.
